# tRNA anticodon cleavage by target-activated CRISPR-Cas13a effector

**DOI:** 10.1126/sciadv.adl0164

**Published:** 2024-04-24

**Authors:** Ishita Jain, Matvey Kolesnik, Konstantin Kuznedelov, Leonid Minakhin, Natalia Morozova, Anna Shiriaeva, Alexandr Kirillov, Sofia Medvedeva, Alexei Livenskyi, Laura Kazieva, Kira S. Makarova, Eugene V. Koonin, Sergei Borukhov, Konstantin Severinov, Ekaterina Semenova

**Affiliations:** ^1^ Waksman Institute for Microbiology, Rutgers, The State University of New Jersey, Piscataway, NJ, USA.; ^2^ Center for Molecular and Cellular Biology, Skolkovo Institute of Science and Technology, Moscow, Russia.; ^3^ Peter the Great St. Petersburg Polytechnic University, Saint Petersburg, Russia.; ^4^ Saint Petersburg State University, Saint Petersburg, Russia.; ^5^ Center for Precision Genome Editing and Genetic Technologies for Biomedicine, Institute of Gene Biology, Moscow, Russia.; ^6^ Faculty of Bioengineering and Bioinformatics, Lomonosov Moscow State University, Moscow, Russia.; ^7^ Institute of Biomedical Chemistry, Moscow, Russia.; ^8^ National Center for Biotechnology Information, National Library of Medicine, National Institutes of Health; Bethesda, MD, USA.; ^9^ Department of Cell Biology and Neuroscience, Rowan University School of Osteopathic Medicine at Stratford; Stratford, NJ, USA.

## Abstract

Type VI CRISPR-Cas systems are among the few CRISPR varieties that target exclusively RNA. The CRISPR RNA–guided, sequence-specific binding of target RNAs, such as phage transcripts, activates the type VI effector, Cas13. Once activated, Cas13 causes collateral RNA cleavage, which induces bacterial cell dormancy, thus protecting the host population from the phage spread. We show here that the principal form of collateral RNA degradation elicited by *Leptotrichia shahii* Cas13a expressed in *Escherichia coli* cells is the cleavage of anticodons in a subset of transfer RNAs (tRNAs) with uridine-rich anticodons. This tRNA cleavage is accompanied by inhibition of protein synthesis, thus providing defense from the phages. In addition, Cas13a-mediated tRNA cleavage indirectly activates the RNases of bacterial toxin-antitoxin modules cleaving messenger RNA, which could provide a backup defense. The mechanism of Cas13a-induced antiphage defense resembles that of bacterial anticodon nucleases, which is compatible with the hypothesis that type VI effectors evolved from an abortive infection module encompassing an anticodon nuclease.

## Teaser

*Leptotrichia shahii* Cas13a specifically cleaves tRNAs upon target RNA recognition, thus ensuring antiphage defense.

## INTRODUCTION

Among the numerous, evolutionarily and mechanistically diverse CRISPR-Cas systems, type VI stands out for the exclusive targeting of RNA by the single-subunit effector ribonuclease (RNase) Cas13 (*1*–*4*). In laboratory experiments, type VI CRISPR-Cas systems provide efficient protection against RNA phages (*2*) as well as against DNA phages, the dominant component of bacterial viromes, via phage transcript recognition, which results in dormancy induction in infected cells (*5*, *6*). Target RNA binding by Cas13 programmed with a cognate CRISPR RNA (crRNA) triggers a conformational rearrangement of the two higher eukaryotes and prokaryotes nucleotide–binding (HEPN) domains of Cas13, which form an active nuclease that catalyzes collateral cleavage of noncomplementary RNAs (*2*, *7*–*9*). Collateral RNA cleavage was observed after target recognition in vitro and is considered to be nonspecific (*2*, *8*, *9*). In *Escherichia coli*, targeting of nonessential transcripts by *Leptotrichia shahii* Cas13a slowed down cell growth, which was proposed to be a consequence of collateral degradation of essential transcripts (*2*). Targeting phage transcripts by *Listeria* Cas13a caused massive degradation of both host and phage RNAs and induced cell dormancy, preventing the spread of phage progeny through the bacterial population (*5*).

Presently, it is unclear whether the target-activated Cas13a RNase activity is solely and directly responsible for dormancy induction, and if so, what are the main, essential substrates of Cas13a RNase, or whether Cas13a functions by activating other cellular stress response systems. In this work, we investigated the collateral activity of Cas13a from *L. shahii* and determined that this protein (LshCas13a), when activated by the recognition of target RNAs, specifically cleaves tRNAs at anticodon loops in vivo in a heterologous *E. coli* host and in vitro. In *E. coli* cells expressing the *L. shahii* CRISPR-Cas13a system, tRNA cleavage by target-activated Cas13a leads to protein synthesis inhibition and slows down cell growth. In addition, collateral tRNA cleavage by Cas13a indirectly activates bacterial stress response systems including RNases encoded by toxin-antitoxin modules. In particular, targeting of a transcript of a DNA phage by LshCas13a induces tRNA cleavage and limits infection. Together, these results reveal the mechanism of type VI CRISPR-Cas immunity.

## RESULTS

### Cas13a RNA targeting slows down cell growth by inhibiting protein synthesis

**Fig. 1.**
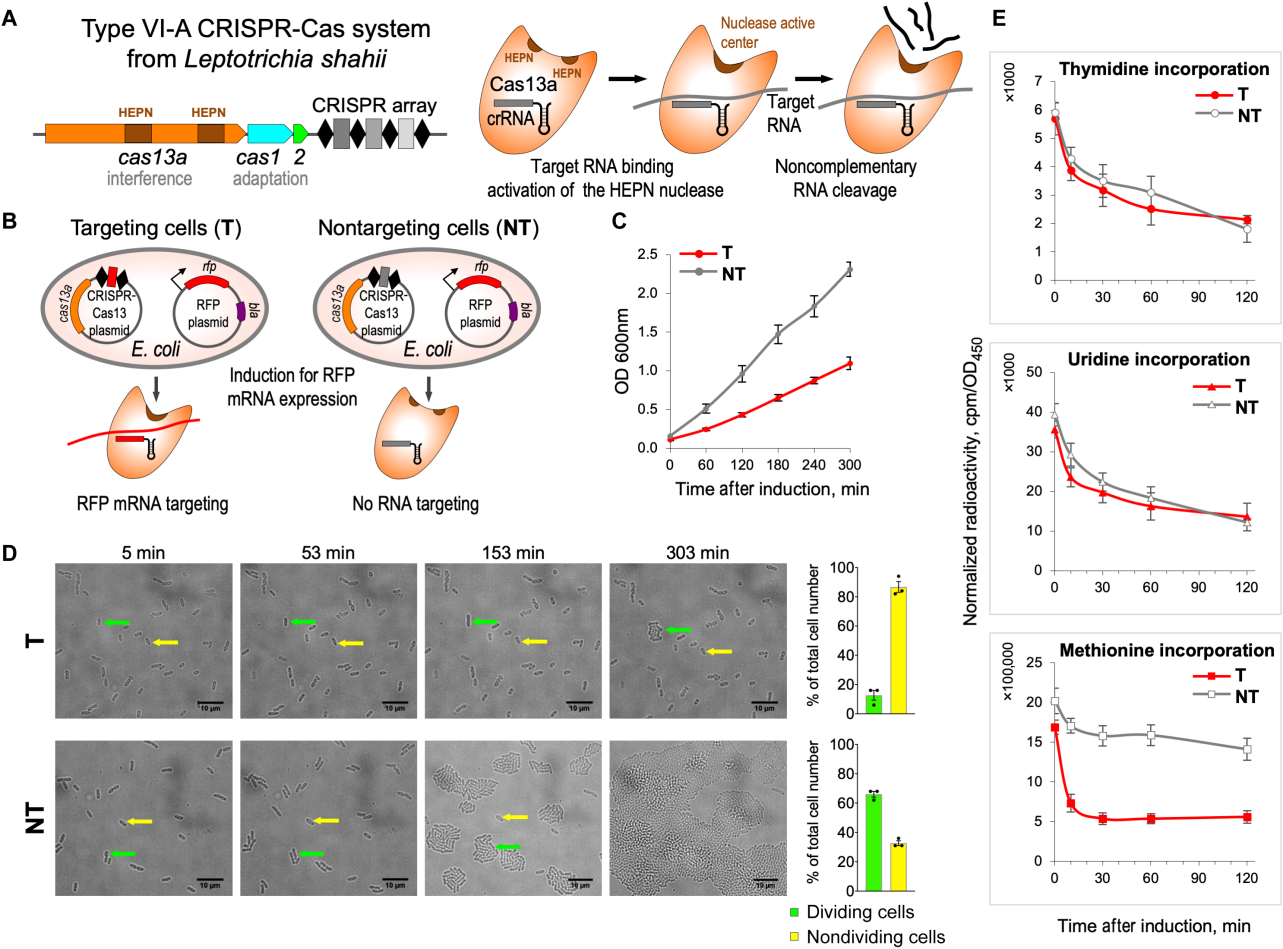
Cell growth is retarded and translation is inhibited in *E. coli* cells harboring LshCas13a activated by a nonessential target RNA. (**A**) Schematic of the *L. shahii* CRISPR-Cas13a locus (left) encoding the Cas13a protein with two HEPN domains involved in interference, the Cas1 and Cas2 adaptation proteins, and a CRISPR array. Cas13a collateral RNase activity (right): Target RNA binding to cognate crRNA activates the HEPN nuclease leading to cleavage of noncomplementary RNA. (**B**) RFP mRNA targeting by LshCas13a in *E. coli*. Cells contain the CRISPR-Cas13 plasmid that encodes LshCas13a and crRNA and the RFP plasmid that directs inducible expression of RFP. In targeting cells (T), CRISPR array contains a spacer (shown in red) matching a site in RFP mRNA; in nontargeting control cells (NT), the CRISPR array spacer (shown in gray) does not match any cellular RNA. (**C**) Targeting of nonessential RFP mRNA by LshCas13a leads to growth retardation in targeting (T, red) cells; no such effect is observed in nontargeting (NT, gray) control. OD_600_ (optical density at 600 nm) values represent means ± SEM of three biological replicates. (**D**) Time-lapse microscopy showing dividing (green arrows) and nondividing (yellow arrows) cells. Bar graph shows mean percentages (± SEM, *n* = 3) of dividing and nondividing cells from the total number of cells counted for targeting (top graph) and nontargeting (bottom graph) samples. At least 100 cells were analyzed for each experiment. (**E**) Time course showing incorporation of radiolabeled precursors: [^3^H]-thymidine, [^3^H]-uridine, or [^35^S]-methionine by pulse labeling in targeting and nontargeting *E. coli* cells. Radioactivity at each time point is normalized to OD_450_ of the samples taken after RFP induction. Means ± SEM, *n* = 3 are shown.

Throughout this work, we used a previously described experimental model of *E. coli* cells transformed with a plasmid containing the *L. shahii* type VI-A CRISPR-Cas system targeting a red fluorescent protein (RFP) mRNA, which is inducibly transcribed from a compatible plasmid (*2*). We will refer to these cells as “targeting” cells. As a control, we included “nontargeting” cells containing a type VI-A CRISPR array encoding crRNAs carrying spacers that did not match sequences in the *E. coli* genome or in the plasmids used in the experiments (Fig. 1, A and B). In agreement with a previous report (*2*), targeting the nonessential RFP transcript substantially decreased the rate of cell growth (Fig. 1C). This effect did not depend on the presence of an antibiotic essential for the selective maintenance of the plasmid encoding the target RNA (fig. S1).

Time-lapse microscopy showed that targeting RFP mRNA by the *L. shahii* type VI-A CRISPR-Cas system did not lead to cell lysis or changes in cell morphology but markedly decreased the proportion of dividing cells in the culture, while most control nontargeting cells divided multiple times during the 300-min post-induction observation and formed microcolonies (Fig. 1D). Cell viability was tested using membrane-impermeable DNA binding dye YOYO-1 (fig. S2). The membranes of nondividing targeting cells remained intact, and only a small (<2%) fraction of cells was dead 180 min after induction. A similar fraction of dead cells was observed in the nontargeting culture (fig. S2). Although the observed unchanged morphology and membrane integrity of nondividing cells are not sufficient to prove the state of dormancy, we now favor an idea that targeting of a nonessential RNA by Cas13a likely induces dormancy rather than cell death. This parallels observations in *Listeria* cells targeting phage transcripts (*5*) and is consistent with a presumed function of the *L. shahii* type VI-A CRISPR-Cas system in adaptive immunity.

To determine which major cellular processes were affected by Cas13a RNA targeting, we performed pulse-labeling analysis using radiolabeled precursors of replication, transcription, and translation. [^3^H]-thymidine and [^3^H]-uridine incorporation rates were comparable in targeting and nontargeting cells cultures, indicating that target recognition by Cas13a had no major effects on DNA replication or RNA synthesis during transcription. In contrast, [^35^S]-methionine incorporation was strongly inhibited in the targeting but not nontargeting cells cultures, indicating that protein synthesis was affected (Fig. 1E). Analysis of radioactively labeled proteins synthesized post-induction by SDS–polyacrylamide gel electrophoresis (PAGE) indicated that translation inhibition was not specific to RFP, whose mRNA was targeted by Cas13a. Instead, decreased synthesis of all detectable cellular proteins was apparent as early as 5 min after induction (fig. S3).

### mRNAs and tRNAs are cleaved at specific sites in targeting cells

To investigate the cause(s) of global translation inhibition observed in targeting cells, total RNA isolated 60 min after induction was subjected to RNA sequencing (RNA-seq), and normalized 5′-end counts of mapped transcripts at each nucleotide position were compared for targeting and nontargeting samples. Overrepresented 5′-end counts were considered an indication of potential cleavage sites. Because collateral RNA degradation might cause global changes in the *E. coli* transcriptome, we analyzed transcription start sites (TSSs) to rule out the possibility that overrepresented 5′-end counts detected in targeting samples corresponded to TSSs activated by Cas13a RNA targeting rather than cleavage sites. The top 1000 TSSs (sorted by adjusted *P* values) predicted in targeting samples were compared with the top 1000 predicted RNA cleavage sites. Only five sites were shared between those two sets, suggesting that the emergence of additional TSSs made little if any contribution to the observed 5′-end enrichment between targeting and nontargeting samples and, therefore, validating the approach for identification of cleavage sites (see Supplementary Text for TSS analysis).

**Fig. 2.**
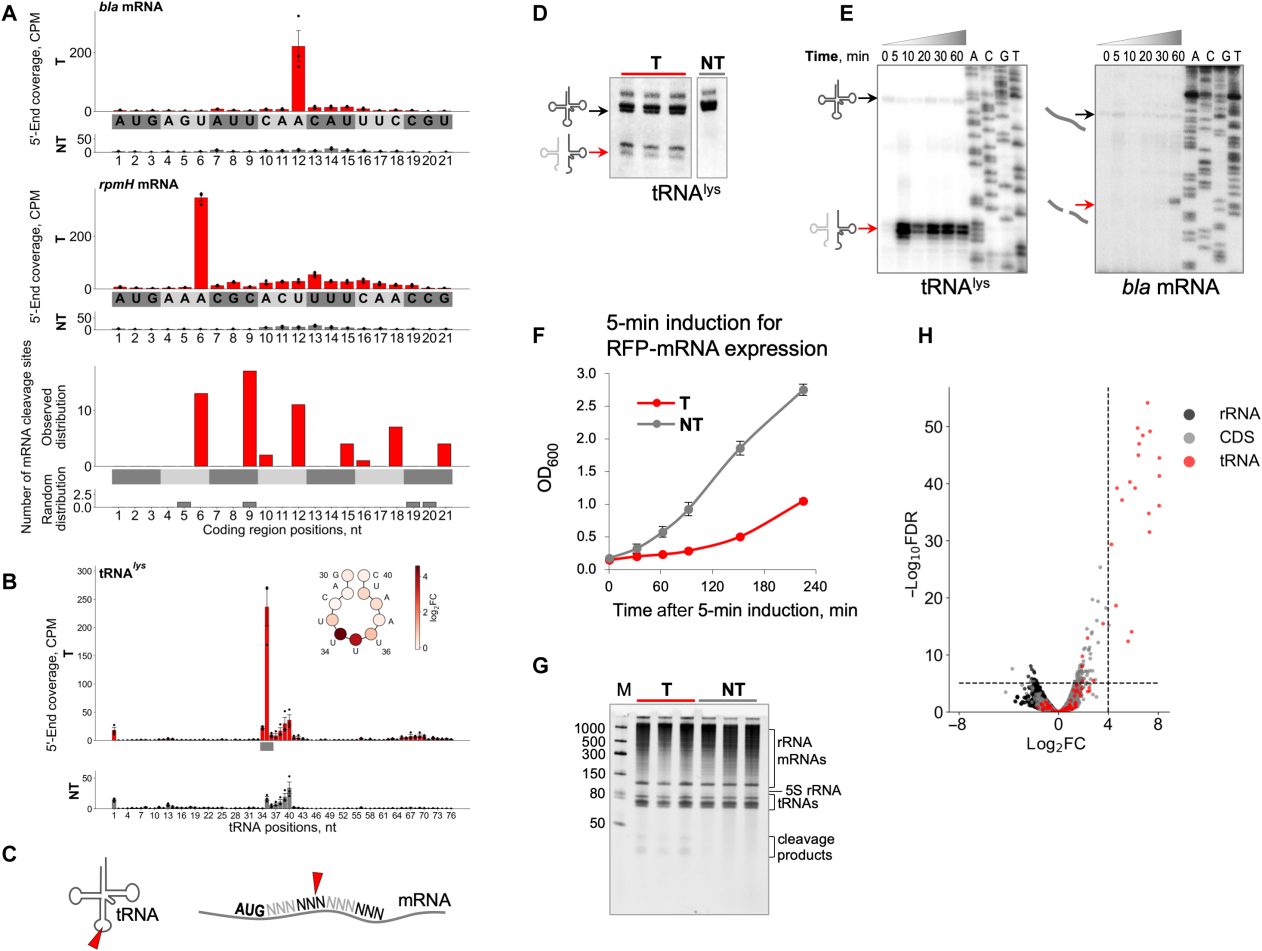
Cas13a-mediated RNA cleavage in targeting cells. (**A**) CPM (counts per million) coverage of RNA 5′ ends mapped to coding regions of *bla* (top) and *rpmH* (middle) genes in targeting (T, red bars) and nontargeting (NT, gray bars) samples of *E. coli* cells at 60 min after induction (means ± SEM, *n* = 3). The bottom panel depicts the distribution of top 100 of RNA cleavage sites. Codons are shown in gray/dark-gray colors. (**B**) 5′-End coverage mapped onto tRNA^lys^ gene (means ± SEM, *n* = 3). Anticodon is shown by a gray bar. At the scheme of tRNA^lys^ anticodon loop, each circle presenting tRNA nucleotide is colored according to values of log_2_ fold change (log_2_FC) between mean 5′-end CPMs in targeting and nontargeting samples. (**C**) Schematic representation of tRNA and mRNA cleavages. (**D**) Northern blot quantification of tRNA^lys^ cleavage. Three biological replicates of targeting and one replicate of nontargeting cells are shown. (**E**) Kinetics of cleavage products accumulation for tRNA^lys^ and *bla* mRNA revealed by primer extension assay. (**F**) Cell growth after 5-min pulse expression of target RNA (means ± SEM, *n* = 3). (**G**) Total RNA isolated at 5 min after induction was resolved by denaturing gel electrophoresis followed by SYBR Gold staining. RNA samples from three biological replicates are shown. (**H**) Volcano plot depicting the results of the comparison of 5′-end counts between targeting and nontargeting *E. coli* samples 5 min after induction using likelihood ratio test. Each dot corresponds to analyzed nucleotide position. The horizontal axis depicts log_2_FC value between targeting and nontargeting samples; the vertical axis depicts adjusted *P* value [false discovery rate (FDR)]. Top 100 of 5′ transcript ends counts (sorted by adjusted *P* values) are shown above the horizontal dashed line. The threshold value of log_2_FC = 4 is indicated by the vertical dashed line.

Numerous cleavages in RNAs isolated from targeting cells were identified and mapped on *E. coli* and plasmid transcripts. The results showed that many mRNAs were prominently cut between the second and third nucleotides of codons located in the beginning of coding regions (Fig. 2A and fig. S4, A and B). As a representative result, Fig. 2A shows the specific cleavage of plasmid-encoded *bla* transcript and genomic *rpmH* transcript at the fourth and second codons, respectively. The observed 3-nucleotide (nt) periodicity of mRNA cleavage sites distribution along the coding regions suggested that cleavage could be coupled to the elongation of translation (Fig. 2A).

In addition to mRNA cleavage, pronounced cleavage of tRNAs at the anticodon loops was detected in targeting cells (Fig. 2, B and C). The extent of cleavage varied for different tRNAs, with some, in particular, lysine and glutamine tRNAs, being cut in measurable amounts, whereas others remaining largely intact (fig. S5). However, none of tRNAs appeared to be completely eliminated by cleavage in the targeting cells. Quantification of the extent of cleavage by Northern blot hybridization showed that only modest fractions of specific tRNA were cleaved (22.8 ± 1.6% of tRNA^lys^, 9.2 ± 2.4% of tRNA^gln^, and 10.6 ± 0.9% of tRNA^leu^; means ± SEM, *n* = 3; Fig. 2D and fig. S6). Overall, the data suggest that the cleavage of mRNAs and tRNAs is responsible for the inhibition of protein synthesis and slower growth upon Cas13a RNA targeting.

A common feature of type VI effectors is the presence of two HEPN domains required for RNase activity (*1*). Each HEPN domain contains a catalytic dyad of conserved arginine and histidine residues (*2*, *10*). Substitutions of any of these residues abolish the Cas13 RNase activity in vitro and protection against RNA phages in vivo (*2*). Cultures of targeting cells expressing Cas13a with single–amino acid substitutions in the HEPN catalytic residues grew at the same rate as nontargeting control cultures (fig. S7, A to C). As expected, HEPN mutations had no effect on crRNA processing, which is known to depend on a distinct RNase active site of Cas13a (fig. S7D) (*8*, *11*). In contrast, primer extension analysis showed that inactivation of the HEPN RNase abolished target-activated tRNA and mRNA cleavage (fig. S7E). Thus, Cas13a HEPN RNase is responsible, directly or indirectly, for the RNA cleavage observed in targeting cells.

### tRNA cleavage precedes mRNA cleavage

The kinetics of accumulation of tRNA and mRNA cleavage products differed substantially (Fig. 2E). While tRNA cleavage was readily detectable as early as 5 min after induction, consistent with rapid protein synthesis inhibition (fig. S3), mRNA cleavage products accumulated much later and became detectable ~60 min after induction. Such different cleavage kinetics suggested that mRNA cleavage could be a consequence of tRNA cleavage and/or translation inhibition followed by activation of cellular RNases. To differentiate between the primary and secondary effects of Cas13a-mediated activity, we performed pulse expression of target RNA. RFP mRNA transcription was induced for 5 min, then the inducer was washed, and growth in the absence of induction was monitored. The transient expression of target RNA was sufficient for growth retardation (Fig. 2F). No signs of global RNA damage were detected in RNA samples isolated 5 min after induction. In contrast, RNA fragments of less than 50 nt that presumably reflect tRNA cleavage were detected in targeting cells (Fig. 2G). RNA-seq revealed tRNA but not mRNA cleavage in targeting samples 5 min after induction (Fig. 2H and table S1).

### tRNA cleavage is unaffected in targeting cells lacking RNase toxins

**Fig. 3.**
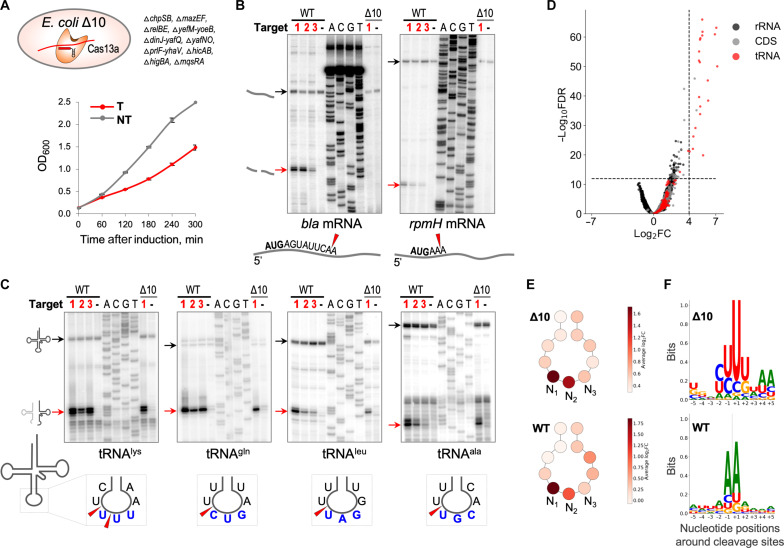
tRNAs but not mRNAs are cleaved in targeting cells lacking 10 toxin RNases. (**A**) RFP mRNA targeting by LshCas13a expressed in *E. coli* Δ10 results in growth retardation (T, red); no such effect is observed in nontargeting (NT, gray) control, same as shown in wild-type *E. coli* (Fig. 1C), means ± SEM, *n* = 3. (**B**) Primer extension assay revealed mRNA cleavage in the presence of toxin RNases, but not in *E. coli* Δ10. (**C**) tRNA cleavages are intact in the cells lacking toxin RNases. Black arrows depict the position of full-size RNA, and red arrows depict the position of cleavage product. mRNA and tRNA cleavages were analyzed in wild-type *E. coli* cells expressing LshCas13a-crRNAs targeting three different positions in RFP mRNA shown as targets 1, 2, and 3 (details in fig. S8), and Δ10 cells expressing LshCas13a-crRNA1 corresponding to target 1 in RFP mRNA. (**D**) Volcano plot depicting the results of the comparison of 5′-end counts per nucleotide position of each strand between targeting and nontargeting samples of Δ10 *E. coli* strain. The analysis was performed in the same way as for Fig. 2H. (**E**) Schematic depiction of tRNA anticodon loop; circles correspond to tRNA nucleotides. Each circle is colored according to the average value of log_2_FC between average 5′-end CPMs in targeting and nontargeting samples across all tRNA genes detected in *E. coli* genome. N_1_, N_2_, and N_3_ nucleotides correspond to tRNA anticodon. (**F**) Weblogo plots built from 10-nt sequences surrounding top 100 of RNA cleavage sites. The position of cleavage is depicted by dash lines. In E and F, the top (Δ10) and the bottom [wild type (WT)] panels correspond to the experiments with Δ10 and wild-type *E. coli* strains, respectively.

The cleavage of mRNA observed in targeting cells 60 min after induction could be induced by RNase toxins. To further elucidate the potential impact of mRNA cleavage in Cas13a-mediated cell growth retardation, we used Δ10, an *E. coli* strain that lacks 10 endoribonuclease-encoding type II toxin-antitoxin systems (*12*). Wild-type and Δ10 cells were transformed with a plasmid for the inducible expression of RFP and the targeting or nontargeting variant of the *L. shahii* type VI-A CRISPR-Cas system plasmid. Retardation of the Δ10 culture growth was observed upon induction of RFP mRNA targeting (Fig. 3A). RNA cleavage was next monitored by primer extension. In wild-type cells, mRNA (Fig. 3B) and tRNA (Fig. 3C) cleavages at the same RNA sites were observed in cells expressing crRNAs that targeted three different positions of the RFP transcript (fig. S8), confirming that it was target recognition as such that triggered mRNA and tRNA degradation. Primer extension revealed no cleavage of selected mRNAs in RNA prepared from the Δ10 targeting culture, suggesting that some of the deleted toxins were responsible for mRNA cleavage observed upon Cas13a RNA targeting in wild-type cells (Fig. 3B). In contrast, tRNAs were efficiently cleaved in the Δ10 samples (Fig. 3C). RNA purified from the Δ10 samples was also analyzed by RNA-seq. Massive tRNA cleavage with a pattern similar to that observed in RNA from wild-type cells was detected (Fig. 3, D and E, table S2, and fig. S5). In both cases, tRNAs were cut mainly at first anticodon nucleotides (Fig. 3E and fig. S9). The consensus of the top 100 cleavage sites detected in Δ10 cells (sorted by adjusted *P* values) demonstrated a preference for uridine residues, in contrast to the adenine-rich consensus of the top 100 cleavage sites from the wild-type cells (Fig. 3F). Previous analysis of LshCas13a collateral damage specificity in vitro also detected a marked preference for uridine residues at the cleavage site (*2*). Thus, the A-rich cleavage site consensus in targeting wild-type *E. coli* cells reflects the preferences of the toxin RNases rather than the LshCas13a cleavage preference.

The genome and the resident plasmid of *L. shahii* encode six type II toxin-antitoxin systems (*13*). These include RelE-, MazF-, YoeB-, and YafQ-like toxin RNases (table S3) related to those present in wild-type *E. coli* and deleted in the Δ10 strain used in our experiments. Thus, the observed indirect activation of *E. coli* toxin RNases upon Cas13a RNA targeting is likely not limited to the heterologous *E. coli* system but could also occur in native *L. shahii* cells.

### Cas13a RNA targeting limits DNA phage infection and causes tRNA cleavage

**Fig. 4.**
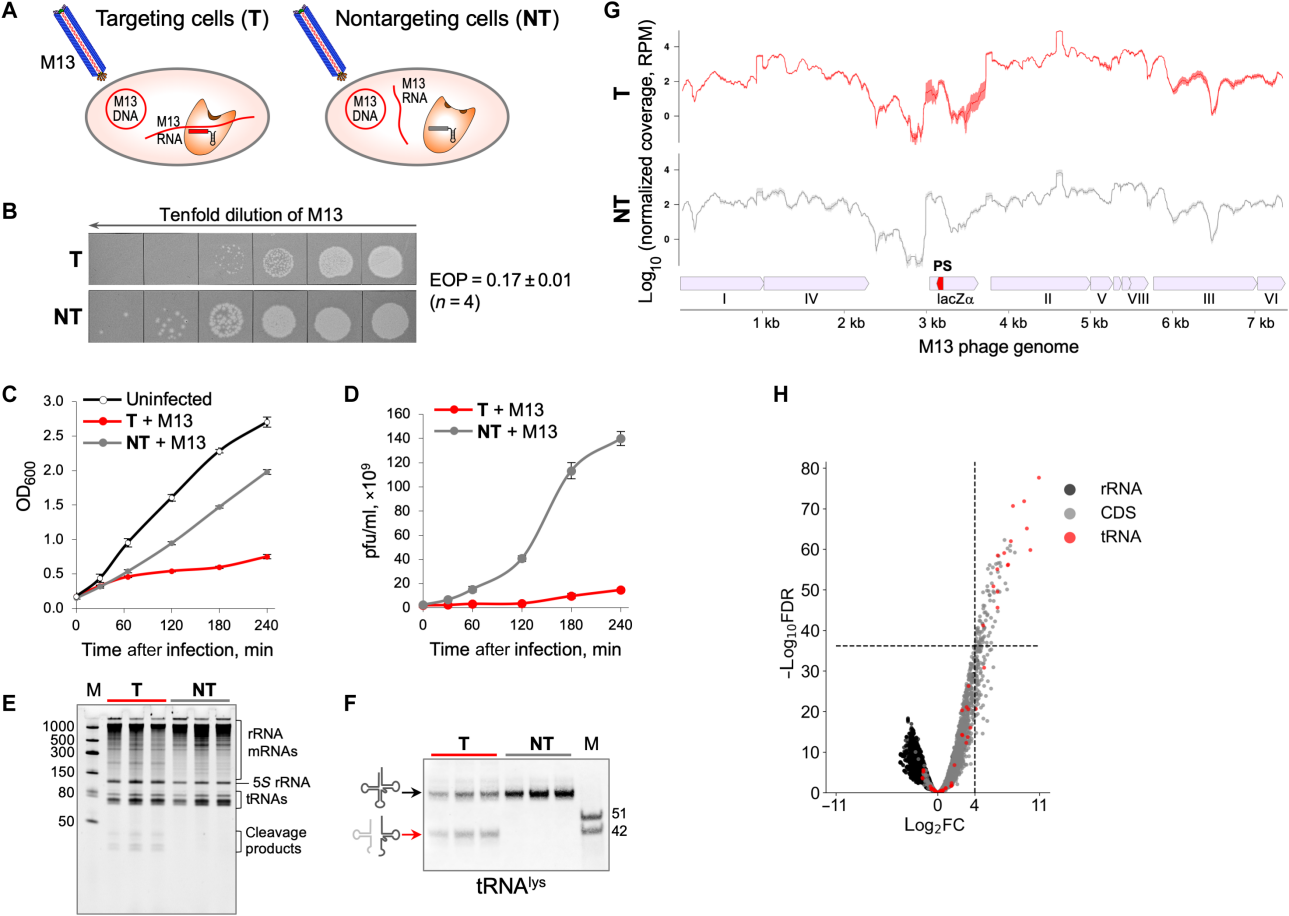
Targeting of a nonessential M13 phage transcript induces tRNA cleavage in infected cells and prevents the infection spread. (**A**) Schematic of targeting by LshCas13a of M13 phage transcript in *E. coli*. Targeting cells (T) express crRNA with a spacer (shown in red) matching M13 RNA, and, in nontargeting control cells (NT), the crRNA spacer (shown in gray) does not match any RNA. (**B**) Plaque assay of 10-fold serial dilutions of M13 lysate spotted on lawns of targeting or nontargeting cells. The efficiency of plaquing (EOP) is presented as means ± SEM, *n* = 4. (**C**) Targeting (red curve) and nontargeting (gray curve) cells in liquid cultures were infected with M13 phage at OD_600_ = 0.2 and multiplicity of infection = 10, and growth rate was monitored along with uninfected control (means ± SEM, *n* = 3). (**D**) Phage titers [plaque-forming units (pfu)/ml] in infected cell cultures measured at indicated time points after infection (means ± SEM, *n* = 3). (**E**) Total RNA isolated from M13-infected cells at 30 min after infection was resolved by denaturing gel electrophoresis followed by SYBR Gold staining. RNA samples from three biological replicates of targeting and nontargeting cells are shown. (**F**) Northern blot quantification of tRNA^lys^ cleavage. (**G**) RNA-seq data show similar patterns of normalized read coverage [reads per million (RPM)] mapped on M13 phage genome for RNA isolated from targeting and nontargeting M13-infected cells. Small red pentagon indicates the target protospacer site within the *lac*Zα gene. Line and shaded error bar represent means ± SEM of three biological replicates. (**H**) Volcano plot depicting the results of the comparison of 5′-end counts per nucleotide position of each strand between targeting and nontargeting samples of M13-infected cells. The analysis was performed in the same way as for Fig. 2H.

Because the principal function of CRISPR-Cas systems is antiviral defense, we investigated the activity of Cas13a in phage-infected cells. Previously, it was demonstrated that LshCas13a restricts infection of MS2, an RNA phage (*2*). Here, we used LshCas13a to target a nonessential transcript of M13, a DNA phage (Fig. 4A). M13 produced fewer plaques on lawn of targeting cells compared to nontargeting control [efficiency of plaquing (EOP), 0.17 ± 0.01, means ± SEM, *n* = 4). Moreover, the infection of targeting cells resulted in a reduced plaque size (Fig. 4B). Thus, RNA targeting by Cas13a interferes with M13 infection. As expected, in the absence of RNA targeting, the chronic M13 infection in liquid medium led to a moderate decrease in growth rate and efficient production of progeny phage particles (Fig. 4, C and D; M13 infection of nontargeting cells shown in gray). In contrast, Cas13a-targeting resulted in growth retardation and suppression of progeny phage production (Fig. 4, C and D; M13 infection of targeting cells shown in red). Thus, M13 phage propagation is impaired when Cas13a targets a phage transcript.

Total RNA isolated from targeting and nontargeting cells 30 min after M13 infection was analyzed by denaturing gel electrophoresis (Fig. 4E). Similar to results obtained for plasmid RNA targeting (Fig. 2G), no signs of overall RNA degradation were observed in targeting and nontargeting samples. However, prominent RNA fragments of less than 50 nt were present in RNA from targeting samples. Northern blot hybridization with a probe complementary to tRNA^lys^ confirmed Cas13a-mediated cleavage at the anticodon loop (Fig. 4F). The extent of tRNA^lys^ cleavage was 37.3 ± 3.3% (means ± SEM, *n* = 3). RNA-seq results showed that M13 RNA targeting did not induce degradation of phage transcripts (Fig. 4G). Analysis of RNA cleavage sites showed that the most common cleavages occurred in anticodon loops (Fig. 4H and table S4). Notably, the subset of tRNAs cleaved in M13-infected cells mirrored that in cells where plasmid RNA was targeted (Fig. 2H and table S1, and Fig. 3D and table S2). Analysis of non-tRNA cleavage sites in M13-infected cells did not detect any particular sequence or secondary structure preferences.

### Target-activated LshCas13a cleaves tRNAs at the anticodon loop in vitro

**Fig. 5.**
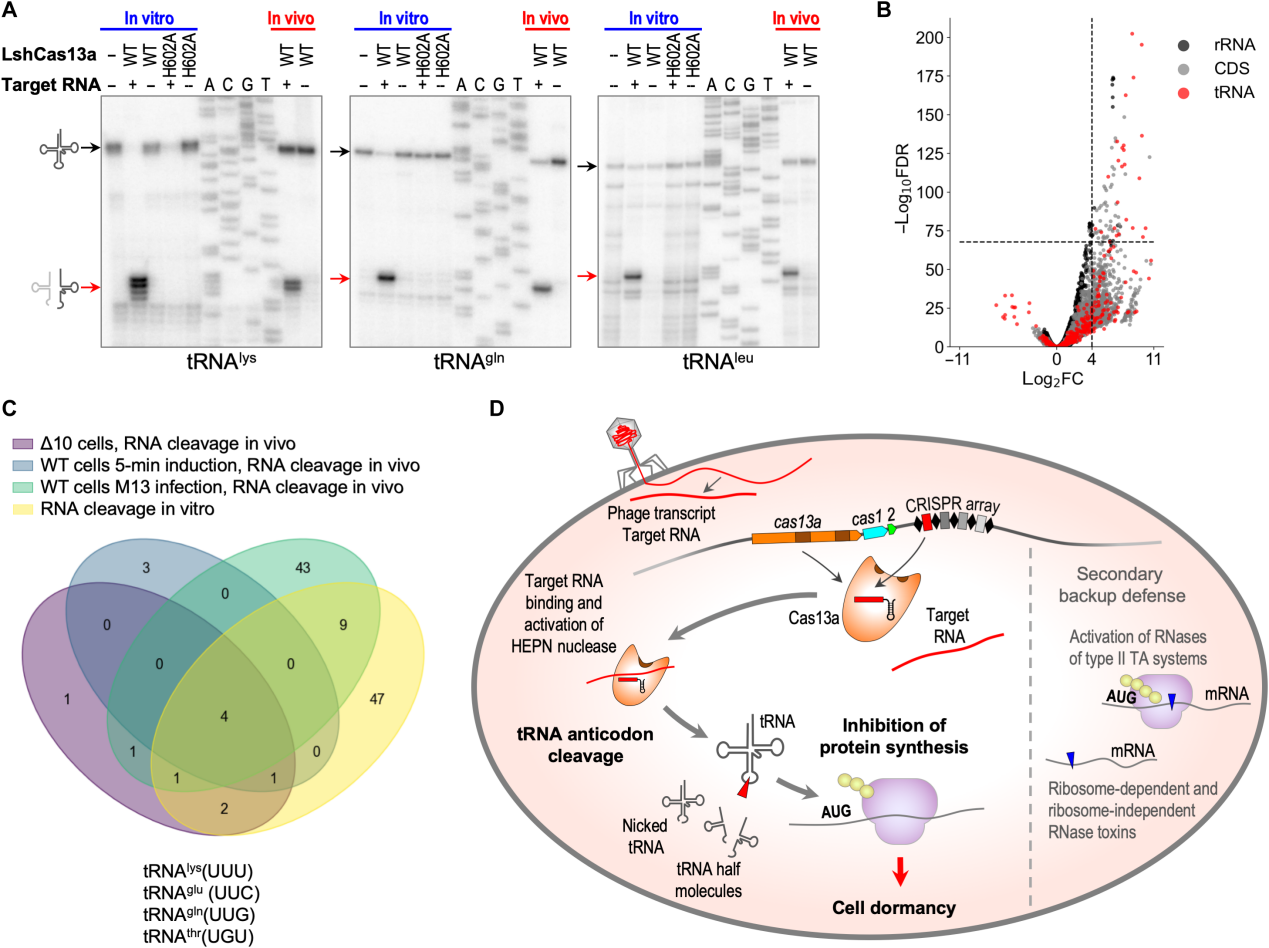
tRNA cleavage is the prime mechanism of CRISPR-Cas immunity in type VI-A system of *L. shahii*. (**A**) Target-activated Cas13a cuts tRNA at the anticodon loop in vitro. Bulk tRNA was used as a substrate for cleavage. Cleavage products revealed by primer extension following in vitro cleavage by target-activated LshCas13a match those observed in vivo. As expected, the H602A mutation of the catalytic residue in the HEPN1 domain abolishes LshCas13a cleavage activity. Black arrows show full-size tRNAs, and red arrows indicate cleavage products. (**B**) Volcano plot depicting the results of the comparison of 5′-end counts per nucleotide position of each strand between targeting and nontargeting samples in in vitro cleavage experiments with isolated total *E. coli* RNA. The analysis was performed in the same way as for Fig. 2H. (**C**) tRNA^lys^, tRNA^glu^, tRNA^gln^, and tRNA^thr^ are the primary substrates for target-activated Cas13a cleavage in vivo and in vitro. Venn diagram shows the overlaps between the sets of RNA cleavage sites detected by RNA-seq from the four experimental systems used in this work: targeting of plasmid encoded RFP mRNA in wild-type (5 min after induction) and Δ10 (60 min after induction) *E. coli* cells; phage RNA targeting in M13-infected cells; and RNA cleavage by LshCas13a in vitro. For each dataset, 5′ transcript ends counts with log_2_FC value between targeting and nontargeting samples > 4 were selected from top 100 hits sorted by adjusted *P* values in ascending order. Resulting sets of RNA cleavage sites were used to build Venn diagram. (**D**) Model of defense provided by CRISPR-Cas13a system of *L. shahii*.

Given that *E. coli* K12 used throughout this work as the host strain does not encode known anticodon nucleases, we hypothesized that tRNAs were cleaved directly by Cas13a activated by the interaction with its RNA target. To test this hypothesis, we performed an in vitro cleavage assay followed by primer extension analysis to characterize the cleavage products (fig. S10A). Recombinant LshCas13a loaded with crRNA was combined with the corresponding target RNA and supplemented with bulk *E. coli* tRNA; nontargeting control reactions were performed in the absence of the target RNA. Cleavage of tRNAs at the same anticodon loop sites as observed in vivo was detected in the presence of target RNA, and a mutation of a HEPN catalytic residue abolished this cleavage (Fig. 5A). We next asked whether LshCas13a could specifically cleave tRNA in vitro in the presence of other cellular RNAs. We repeated the cleavage assay using total *E. coli* RNA as the cleavage substrate and detected the same tRNA cleavage products (fig. S10B). Thus, LshCas13a is an anticodon tRNase that is activated by target recognition and is active even in the presence of an excess of other RNAs.

Total RNA was used as the substrate for in vitro cleavage by LshCas13a followed by RNA-seq to test whether target-activated Cas13a cleaved RNAs other than tRNAs. Similar to the in vivo results, we detected massive tRNA cleavage at the anticodon loops (Fig. 5B and figs. S10C and 11) as well as some cleavage of mRNAs and rRNAs (Fig. 5B). A closer examination of the minor non-tRNA cleavage products showed that LshCas13a cut them at uridine residues located in small loops, apparently mimicking the anticodon stem-loop structure of tRNA (fig. S12). However, in cells, the abundance of these non-tRNA cleavage products was negligible compared to the tRNA cleavage products (Fig. 3D).

Bacterial tRNAs are heavily modified, especially at the anticodon loops (*14*). To determine whether these modifications were essential for the Cas13a cleavage, unmodified synthetic tRNA^lys^ was tested in the in vitro cleavage assay. Unmodified substrate was cut by target-activated LshCas13a at the same position as modified tRNA from cells (fig. S13), suggesting that the specific structure of tRNAs and the anticodon loop sequence rather than base modification determine the specificity of the LshCas13a cleavage.

### Target-activated LshCas13a preferentially cleaves tRNA^lys^, tRNA^glu^, tRNA^gln^, and tRNA^thr^

To detect the primary cleavage substrates for target-activated LshCas13a, we compared the pattern of RNA cleavages revealed by RNA-seq from the four experimental systems. The sets of RNA cleavage sites detected in wild-type and Δ10 *E. coli* cells upon RFP mRNA targeting, in M13-infected cells, and in vitro were compared (Fig. 5C). Cleavages in four tRNAs—tRNA^lys^ (UUU), tRNA^glu^ (UUC), tRNA^gln^ (UUG), and tRNA^thr^ (UGU)—all with anticodons containing at least two uridines, were detected in all four datasets, indicating that these four tRNAs are preferred cleavage substrates for LshCas13a. Other tRNAs containing uridines in their anticodons can also be cleaved by LshCas13a albeit less efficiently (figs. S5, S9, and S11).

To investigate the potential tRNA cleavage by target-activated LshCas13a in native cells, we compared the anticodon loops in tRNAs from *E. coli* and *L. shahii*, specifically focusing on tRNAs identified as preferred cleavage substrates for LshCas13a (fig. S14). We observed a high similarity in the anticodon loop sequences, suggesting that these tRNAs could be natural cleavage substrates for Cas13a in native *L. shahii* cells. Furthermore, the sequences of the anticodon loops in the corresponding human tRNAs are also strongly conserved (fig. S14), suggesting that potential tRNA cleavage should be considered when introducing Cas13 into human cells for biomedical applications.

### tRNA cleavage by LshCas13a does not affect tRNA aminoacylation and induces a RelA-independent pathway

Imbalances among charged (aminoacylated) tRNA amounts can hinder efficient translation. To determine whether aminoacylation was affected upon Cas13a RNA targeting, we developed a liquid chromatography-mass spectrometry (LC-MS)–based method for global analysis of the tRNA aminoacylation level (GATRAL; fig. S15A). This approach allows one to measure the relative amount of each charged tRNA and examine possible changes of tRNA aminoacylation in response to various conditions. First, we validated the GATRAL assay using *E. coli* cells treated with microcin C (McC), a specific inhibitor of aspartyl-tRNA synthetase (*15*). As anticipated, a ~100-fold decrease in the relative amount of aspartyl-tRNA in the McC-treated cells compared to the untreated cells was observed (fig. S15B). Next, we applied GATRAL to check tRNA aminoacylation under the conditions of RFP mRNA targeting by Cas13a. No substantial changes in the relative amount of charged tRNA in targeting cells compared to nontargeting cells were detected despite the observed growth retardation and tRNA cleavage (fig. S15, C to E). However, GATRAL can detect the aminoacyl moiety not only on charged intact tRNA but also on charged tRNA fragments. Given that 80% of tRNA molecules are charged in exponentially growing *E. coli* cells (*16*), there is a possibility that target-activated Cas13a cleaves charged tRNA. This is unlikely to have changed the level of charged tRNA substantially because only modest fractions of specific tRNAs were cleaved as shown by quantitative Northern blot hybridization (Fig. 2D and fig. S6). Therefore, the GATRAL results support the conclusion that Cas13a targeting does not lead to a gross drop in the amounts of charged tRNAs. Moreover, overexpression of four tRNAs that are most strongly affected by Cas13a targeting (tRNA^lys^, tRNA^gln^, tRNA^glu^, and tRNA^leu^) did not offset the growth defect caused by Cas13a targeting (fig. S16). Compared to control, the levels of intact tRNAs were appreciably higher in cells that overproduced tRNAs. This result suggests that growth retardation caused by Cas13a targeting is not due to the depletion of certain tRNA species but rather is caused by a toxic or signaling activity of tRNA fragments that are produced as a result of cleavage.

The observation of unimpaired aminoacylation raises the question of the mechanism of Cas13a-induced growth retardation. When aminoacylation is affected, binding uncharged tRNAs to ribosomes activates the RelA enzyme, resulting in the synthesis of the transcription regulator (p)ppGpp and global reshaping of cell transcription. The RelA-dependent stringent response allows cells to adapt to the stress conditions (*17*). Analysis of the transcription pattern in the cells upon Cas13a targeting did not reveal appreciable changes in the expression levels of genes known to be either up- or down-regulated under stringent response conditions (fig. S17A). Moreover, if the stringent response were involved, then deletion of *relA* would be expected to prevent dormancy induction and instead would be expected to elicit cell death. Cas13a targeting in the Δ*relA* cells affected cell growth similarly to the *relA*^+^ control. Live microscopy also showed that the proportion of cells that stopped dividing upon Cas13a targeting was the same whether RelA was present or not. The proportion of dead cells (as evidenced by membrane-impermeable dye YOYO-1 staining) was also unchanged (fig. S17, B to D). Together, these results suggest that Cas13a RNA targeting induces a RelA-independent pathway of growth retardation.

## DISCUSSION

Our results lead to the following view of *L. shahii* type VI-A immunity (Fig. 5D). In phage-infected cells carrying a crRNA(s) targeting a phage transcript, target recognition activates the HEPN domains of Cas13a enabling collateral RNA cleavage. Target-activated Cas13a cleaves a subset of tRNAs at their anticodon loops resulting in translation inhibition. tRNAs containing U-rich anticodons are preferentially cleaved reflecting the specificity of the activated LshCas13a tRNase (Fig. 5C and fig. S9). Cleavage of tRNAs and/or translation inhibition can indirectly activate type II toxin RNases that cleave mRNA. Degradation of mRNAs by these toxins further compromises translation. However, direct cleavage of tRNAs by target-activated Cas13a is sufficient to inhibit protein synthesis and retard cell growth in the absence of type II toxin RNases. Thus, tRNA cleavage is probably the primary mechanism through which *L. shahii* type VI-A CRISPR-Cas system induces dormancy and limits the spread of phage infection through the population, whereas activation of toxin RNases is a secondary, backup mechanism.

The type VI CRISPR systems are thought to have evolved from an abortive infection (Abi) system containing a HEPN RNase (*3*, *10*, *18*, *19*). Our present results imply that the ancestral Abi nuclease functioned by abrogating translation via tRNA cleavage. The combination of such a nuclease with a CRISPR array and an adaptation module endowed the system with increased specificity and hence reduced the fitness cost incurred by the toxic Abi module.

Depletion of specific tRNAs leads to ribosome stalling at “hungry” codons, which can trigger frameshift or ribosome bypass and cause the synthesis of aberrant polypeptides (*20*). Some bacterial anticodon nucleases, such as PrrC, apparently cause this outcome because they substantially deplete the pool of particular tRNAs (*21*, *22*). In contrast, in the case of the colicin D anticodon nuclease that specifically cleaves tRNA^arg^, it is the accumulation of cleavage products rather than the depletion of intact tRNA^arg^ that impairs translation (*23*). Our results demonstrate that the Cas13a-mediated tRNA cleavage does not result in the complete depletion of full-size tRNAs, at least, in the surrogate system used here (Figs. 2D and 4F and fig. S6) although the effect apparently sufficed for translation arrest. In addition, the tRNA fragments or nicked tRNAs produced by the Cas13a cleavage might act as signaling or inhibitory molecules. Recently, the signaling function of cleavage products, presumed to be tRNA fragments, generated by Cas13b ortholog, was proposed for the CRISPR-Cas13b–assisted mechanism of antiphage defense through membrane pore activation (*24*).

Bacteriophage RNA ligases rejoin tRNA halves produced by anticodon nucleases, rendering phages resistant to this defense mechanism (*25*, *26*), and, also, the expression of some bacterial tRNA ligases is activated by tRNA fragments (*27*). Such RNA ligases could function as anti-CRISPR mechanisms against type VI, in which case, the activation of RNase toxins would become the primary defense line.

This work specifically focused on *L. shahii* Cas13a but opens avenues for future research into the specificity of various type VI CRISPR-Cas effectors underlying the defense mechanisms. On the practical side, determining the preferred collateral cleavage substrates for target-activated Cas13a effectors could help improve the sensitivity of the powerful SHERLOCK method of nucleic acids detection (*28*) and other applications.

## MATERIALS AND METHODS

### *E. coli* strains and plasmids

*E. coli* C3000 (wild-type) cells were transformed with two plasmids: CRISPR-Cas13 plasmid containing the *L. shahii* (Lsh) Cas13a locus and RFP plasmid for inducible expression of RFP. The targeting cells contained the CRISPR-Cas13a plasmid encoding crRNA spacer targeting the RFP mRNA. The cells contained the CRISPR-Cas13a plasmid encoding crRNAs with no matching sequences in *E. coli* genome and the plasmids were used as the nontargeting control. CRISPR-Cas13 plasmids (pC002, pC003, pC008, and pC003_RFP1) described previously (*2*) were gifts from the F. Zhang lab. Additional CRISPR-Cas13a plasmids were constructed using Golden Gate cloning on the base of pC003 plasmid containing Bsa I sites for spacer cloning (table S5). All CRISPR-Cas13 plasmids have a pACYC184 backbone carrying a chloramphenicol resistance gene. RFP plasmid (pC008) is pBR322 derivative carrying *bla* gene for ampicillin resistance along with *rfp* gene under control of tetR-promoter induced by anhydrotetracycline. *E. coli* Δ10 strain containing genomic deletions of 10 type II toxin-antitoxin systems (Δ*chpSB*, Δ*mazEF*, Δ*relBE*, Δ*yefM-yoeB*, Δ*dinJ-yafQ*, Δ*yafNO*, Δ*prlF-yhaV*, Δ*hicAB*, Δ*higBA*, and Δ*mqsRA*) described previously (*12*) was a gift from the V. Melderen lab. *E. coli* Δ10 strain was transformed with two plasmids: CRISPR-Cas13 plasmid containing the LshCas13a locus encoding Cas13a and crRNA spacer targeting the RFP mRNA, and RFP plasmid for inducible expression of RFP to construct targeting Δ10 cells. In nontargeting Δ10 cells, CRISPR-Cas13 plasmid encodes crRNA spacer with no matching sequences in *E. coli* genome and the plasmids. To assess the role of RelA in LshCas13a-induced growth retardation, *E. coli* strain lacking *relA* gene [Δ*relA*, JW2755 strain from the Keio collection (*29*)] was used in parallel to the parent wild-type strain (BW25113). Both strains were supplemented with corresponding CRISPR-Cas13 and RFP plasmids to get targeting and nontargeting variants. To study the effect of the tRNA expression level on cell growth upon Cas13a RNA targeting, two plasmids expressing tRNA cassettes were generated through gBlock insertion into the derivative pC008 plasmid containing lipoprotein promoter (table S5). These plasmids (fig. S16) provided constitutive expression of tRNA^lys^, tRNA^gln^, tRNA^glu^, and tRNA^leu^ (most strongly affected by target-activated Cas13a) and tRNA^ser^, tRNA^pro^, tRNA^asp^, and tRNA^trp^ (unaffected by target-activated Cas13a) genes under lipoprotein promoter (*30*).

### Cell growth assay

To investigate the effect of the RFP mRNA targeting on the cell growth rate, individual colonies of transformed targeting and nontargeting wild-type or Δ10 *E. coli* cells were grown overnight in LB supplemented with chloramphenicol (25 μg/ml) and ampicillin (100 μg/ml). Cell cultures were diluted 1:100 in fresh LB containing chloramphenicol and ampicillin and grown for 1 hour at 37°C with continuous shaking. After 1 hour, RFP expression was induced with anhydrotetracycline (500 ng/ml) and OD_600_ (optical density at 600 nm) measurements were taken every 30 min. Growth experiments were performed similarly for Δ*relA* (JW2755) and BW25113 strains. For pulse expression of target RNA, RFP mRNA transcription was induced for 5 min, then the cells were washed off the inducer twice with fresh LB, and the cell pellets were suspended in initial volume of LB supplemented with antibiotics. Then, the cell growth was monitored in the absence of induction. All growth experiments were performed at least three times.

### Time-lapse microscopy

For microscopy, *E. coli* C3000 cells supplemented with CRISPR-Cas13 plasmid and RFP plasmid were grown under the same conditions as described above—overnight cultures were diluted 1:100 and grown for 1 hour at 37°C. Aliquots of the cell cultures mixed with 100 nM YOYO-1 dye were dropped on an LB–1.5% agarose block supplemented with chloramphenicol (25 μg/ml) and ampicillin (100 μg/ml), and anhydrotetracycline (500 ng/ml) for induction of RFP expression. Two agarose blocks containing targeting and nontargeting cells were placed into one microscope chamber, and cell growth of two cultures was simultaneous monitored under induced conditions. The experiment was done in triplicates. The Nikon Ti-E inverted microscope was equipped with Andor’s Zyla 4.2 sCMOS camera, Semrock filter Set YFP-2427B for green fluorescence detection and custom-made incubation system to maintain cells at 37°C. Image analysis was done using ImageJ software (*31*). For each of the three replicates, the fate of at least 100 cells were monitored, and two cell types were determined: dividing cells that formed microcolonies over the course of the experiment and nondividing cells that did not form colonies nor had any visible changes in cell morphology. Both cell types were represented as a percentage of the total number of cells counted for each replicate. To distinguish between live and dead cells, green fluorescent membrane-impermeant YOYO-1 dye was used. The dye cannot penetrate live cells but can penetrate dead cells to stain the DNA, making dead cells fluoresce green. A number of dead cells were counted in targeting and nontargeting cultures, and at least 100 cells were analyzed individually for three replicates. A similar analysis was performed for Δ*relA* (JW2755) and wild-type BW25113 strains.

### Metabolic labeling and autoradiography

Targeting and nontargeting *E. coli* C3000 cells were grown in M9 minimal medium containing 18 amino acids without methionine and cysteine till 0.1 OD_600_ at 37°C with continuous shaking. RFP expression was then induced with anhydrotetracycline (500 ng/ml). Aliquots (500 μl) were taken at time 0 and then at 10, 30, 60, and 120 min after induction and mixed with 25 μl of a solution of thymidine (10 μg/ml), uridine (50 μg/ml), or a mixture of methionine and cysteine (10 μg/ml each) containing 1 μCi of radioactive [methyl-^3^H]-thymidine (6.7 Ci/mmol), [5,6-^3^H]-uridine (35–50 Ci/mmol), or L-[^35^S]-methionine (1135 Ci/mmol) (Perkin-Elmer, USA), respectively. Pulse labeling was carried out at 37°C for 2 min with continuous shaking at 300 rpm, followed by the addition of 100 μl of cold 40% trichloroacetic acid (TCA) to stop the reaction. Samples were filtered through glass microfiber filters (GE Healthcare Whatman), and the filters were washed twice with 1 ml of cold 10% TCA and 1 ml of cold 100% ethanol each. The dried filters were placed in 4 ml of scintillation liquid, and the radioactivity was measured in a liquid scintillation counter (LS60001C, Beckman Coulter, USA). Radioactivity counts corresponding to incorporated thymidine, uridine, and methionine were normalized to OD_450_ at each time point. For the analysis of RFP expression relative to overall protein synthesis, targeting and nontargeting cells were grown as described above, except that 500 μl of aliquots for [^35^S]-methionine pulse-labeling reactions were taken before (time 0) and after RFP induction at 2, 5, and 10 min. The samples were analyzed by Mops/SDS 10% PAGE followed by Coomassie staining and quantification by Phosphorimager.

### Targeting M13 phage RNA

To target a nonessential M13 phage transcript, the sequence corresponding to the spacer1 of native CRISPR-Cas13a array of *L. shahii* was cloned into *lacZ*α gene of M13mp18 phage genome. The coding strand of the resulting protospacer was complementary to crRNA containing spacer1. The protospacer was adjacent to cytosine, a functional protospacer flanking sequence. *E. coli* C3000 cells were used as a host for M13 infection. The cells supplemented with the CRISPR-Cas13 plasmid expressing spacer1 crRNA were referred to as targeting cells. The cells that contained the CRISPR-Cas13a plasmid encoding crRNAs containing MS2 phage spacer (*2*) with no matching sequences in *E. coli* genome, M13 phage genome, and the plasmid were used as the nontargeting control. For routine phage propagation and titration, K12F^+^ *E. coli* cells (Novablue, Novagen) were used.

### Phage spot test

To compare the targeting and nontargeting cells’ sensitivity to M13 infection, 10-fold serial dilutions of M13 phage lysate were spotted on the cell lawns. The cells were grown in 2× YT medium supplemented with chloramphenicol (25 μg/ml) at 37°C until OD_600_ reached 0.5. Petri dishes (100 mm by 100 mm) with 2× YT agar supplemented with chloramphenicol (25 μg/ml) were overlaid with 5 ml of soft agar containing 0.5 ml of plating cells suspension. After solidification for 5 min, 5 μl of phage lysate dilutions were spotted on the soft agar surface. Plaque formation was analyzed after overnight incubation. The EOP calculated as a ratio of the numbers of plaque-forming units on the lawns of targeting and nontargeting cells.

### M13 phage infection

Targeting and nontargeting *E. coli* C3000 cells grown in 2× YT medium supplemented with chloramphenicol (25 μg/ml) till 0.2 OD_600_ at 37°C were infected with M13 phage at multiplicity of infection of 10. The infected cells’ growth was monitored by measuring OD_600_ alongside with uninfected cells. Phage development in the infected cells was analyzed by measuring phage titer at indicated time point after infection. All phage experiments were performed at least three times.

### RNA isolation

Total RNA was isolated from *E. coli* cells growing in LB medium and harvested at 60 min after induction of RFP expression. In the experiments with pulse expression of RFP, total RNA was isolated from the cells harvested at 5 min after induction. Cell lysis was done using Max Bacterial Enhancement Reagent (Invitrogen) for 4 min and then with TRIzol reagent (Invitrogen) for 5 min. Total RNA including small RNAs (>17 nt) was purified using a Zymo-Spin column (Direct-zol RNA kit, Zymo Research). RNA was treated with Turbo DNase (Turbo DNA-free kit, Invitrogen) to remove DNA contamination. For RNA purification from M13-infected *E. coli* cells, the cells were harvested at 30 min after M13 phage infection. RNA integrity and cleavage products were analyzed by electrophoresis in 10% polyacrylamide-UREA gel (Novex TBE-UREA Gel 10%, Invitrogen) followed by SYBR Gold staining.

### Primer extension

For primer extension, 3.5 μg of total RNA was reverse-transcribed with the SuperScript IV First-Strand Synthesis System (Invitrogen) according to the manufacturer’s protocol. DNA oligonucleotides (table S6) were radiolabeled at the 5′ end by T4 polynucleotide kinase (PNK) (New England Biolabs) treatment and [γ- ^32^P] (PerkinElmer) for 60 min at 37°C followed by purification on a Micro-Bio Spin P-6 Gel Column (Bio-Rad). One picomol of 5′-end-radiolabeled primer was used for each extension reaction. In parallel, sequencing reactions were performed on amplified polymerase chain reaction (PCR) fragments of genomic or plasmid loci with the corresponding radiolabeled primers using the Thermo Sequenase Cycle Sequencing Kit according to the manufacturer’s instructions. Reaction products were resolved by 10% denaturing PAGE and visualized using a Phosphorimager.

### HEPN mutagenesis

Alanine mutations were created in each of four catalytic residues of LshCas13a HEPN domains (R597A, H602A, R1278A, and H1283A) in plasmid containing LshCas13a locus and a CRISPR array carrying a spacer targeting RFP mRNA (pC003_RFP1) using QuikChange Site-Directed Mutagenesis kit (Agilent) and the mutagenic primers containing the desired mutations (table S6) according to the manufacturer’s protocol. Cell growth experiments were done as described above. Primer extension analysis to determine cleavage products in *bla* mRNA, *rpmH* mRNA, and tRNAs was also done as described above. In all HEPN mutant experiments, wild-type targeting and nontargeting variants were used as controls.

### Northern blot hybridization

The procedure was mostly performed as described before (*32*). To analyze crRNA maturation, 10 μg of total RNA from *E. coli* cells transformed with CRISPR-Cas13 plasmid expressing HEPN mutant LshCas13a proteins with mutations R597A, H602A, R1278A, H1283A, was resolved by 10% denaturing PAGE in Mini Protean 3 Cell (Bio-Rad). Separated RNA was then transferred to a nylon membrane (Hybond-XL, GE Healthcare) in prechilled 0.5× tris-boric acid–EDTA buffer for 90 min at 50 V using Mini Trans-Blot electrophoretic transfer cell (Bio-Rad). Transferred RNA was ultraviolet (UV)–cross-linked to membrane and hybridized with [γ-^32^P] 5′-end labeled RFP-spacer specific probe (RFP_crRNA_probe, table S6) in ExpressHyb solution (Clontech Laboratories Inc.) according to the manufacturer’s protocol. Hybridization was performed in Isotemp rotisserie oven (Thermo Fisher Scientific) for 2 hours at 37°C. Hybridized RNA bands were visualized by phosphorimaging. To detect cleavage of tRNA^lys^, tRNA^gln^, and tRNA^leu^ and to quantify the extent of cleavage, hybridization with a fluorescein-labeled oligodeoxynucleotide probe (Integrated DNA Technologies) specific to the 3′-end of tRNA (table S6) was performed. The extent of tRNA cleavage was quantified on the basis of the intensity of bands corresponding to cleaved tRNA and intact tRNA. The extent of cleavage (%) = 100 × amount of cleaved tRNA fragment/(amount of cleaved tRNA fragment + amount of intact tRNA) (*23*).

### LshCas13a protein purification

Lsh *cas13a* gene was cloned into Nde I and Bam HI sites in pET28 plasmid for protein purification. *E. coli* BL21 (DE3) cells were transformed with pET28_LshCas13a for protein expression and isolation. LshCas13a protein was purified from the cells grown in 400 ml of LB supplemented with kanamycin (50 μg/ml) and 0.5 mM isopropyl-β-d-thiogalactopyranoside (IPTG), as described previously (*33*). Freshly transformed cells were grown at 37°C to OD_600_ of 0.6 to 0.9, then induced with 0.5 mM IPTG, and grown for additional 6 to 8 hours at room temperature before cell harvesting. The cell pellets resuspended in buffer A [20 mM tris-HCl (pH 8.0), 500 mM NaCl, 4 mM imidazole (pH 8.0), 5% (v/v) glycerol, 0.2 mM phenylmethylsulfonyl fluoride] containing protease inhibitor cocktail Roche cOmplete, EDTA-free (Sigma-Aldrich) were lysed by sonication. Lysates were cleared by centrifugation at 15,000*g* for 60 min and filtered through 0.22-μm filter (Millipore) and applied to a 1-ml chelating Hi-Trap Sepharose column (GE Healthcare) equilibrated with buffer A. Proteins were purified first by washing the column using buffer A containing 25 mM imidazole and then eluting with buffer A containing 200 mM imidazole. Pooled protein fractions were diluted 10 times with binding buffer [20 mM tris-HCl (pH 8.0), 5% (v/v) glycerol, 1 mM EDTA, and 2 mM β-mercaptoethanol] and applied to a 1-ml Hi-Trap Heparin column (GE Healthcare) equilibrated with binding buffer. The column was washed with binding buffer containing 500 mM NaCl, and proteins were eluted with binding buffer containing 1 M NaCl. Pooled protein fractions were concentrated using Microsep centrifugal devices 30K (Pall Corp), dialyzed against buffer B [20 mM tris-HCl (pH 8.0), 200 mM NaCl, 50% (v/v) glycerol, 0.5 mM EDTA, and 2 mM β-mercaptoethanol], and then stored at −80°C. HEPN H602A encoding mutation was introduced into pET28_LshCas13a plasmid using a QuikChange Site-Directed Mutagenesis kit as described above, and mutant LshCas13a was purified similar to the wild-type LshCas13a.

### Generation of RNA for in vitro cleavage assay

crRNA and target RNA were transcribed in vitro from PCR-generated double-stranded DNA template using T7 RNA polymerase (New England Biolabs) according to the manufacturer’s recommendations and was purified by electrophoresis in 10% polyacrylamide 6 M urea gels. Oligonucleotide sequences are listed in table S6. Bulk *E. coli* tRNA was ordered from Sigma-Aldrich. Unmodified tRNA^lys^ was ordered synthetically (Integrated DNA Technologies).

### In vitro cleavage assay

In vitro RNA cleavage was performed with LshCas13a at 37°C in cleavage buffer [20 mM tris-HCl (pH 8.0), 100 mM NaCl, 5 mM MgCl_2_, and 1 mM dithiothreitol]. LshCas13a-crRNA complex was formed by combining, in 10 μl, Cas13a and crRNA (200 nM each) and incubating at 37°C for 20 min. Next, 100 nM target RNA was added. Nontargeting control reactions were performed in the absence of target RNA. Immediately after adding target RNA, the reactions were supplemented with collateral RNA. *E. coli* bulk tRNA (0.1 μg), total RNA (2 μg), or unmodified synthetic tRNA^lys^ (0.05 μg) per 10 μl of reaction was used as collateral cleavage substrates. After 60 min of incubation at 37°C, RNA from reaction mixture was extracted with chloroform and precipitated in 75% ethanol in the presence of 0.3 M NaOAc and glycogen (0.1 mg/ml). tRNA cleavage products were analyzed by primer extension as described above. For RNA-seq analysis of cleavage products generated by LshCas13a in vitro, total RNA from *E. coli* cells depleted from rRNA [MICROBExpress Bacterial mRNA Enrichment Kit (Invitrogen)] was used as cleavage substrate, and the cleavage reactions were performed in 40 μl in triplicates.

### RNA sequencing

The general procedure to prepare RNA for sequencing was similar to the protocol described previously (*34*) with some modifications. Total RNA samples were treated with the MICROBExpress Bacterial mRNA Enrichment Kit (Invitrogen) for rRNA depletion before library preparation. To construct libraries that contained both primary transcripts carrying 5′-triphosphate (5′-PPP) and processed transcripts carrying 5′-monophosphate (5′-P) or 5′-hydroxyl (5′-OH), RNA samples were treated with RNA 5′ pyrophosphohydrolase (RppH) (New England Biolabs) for 30 min at 37°C. In parallel, RNA samples without RppH treatment were used for preparation of libraries enriched in processed transcripts. Fragmentation was carried out by sonication using the Covaris protocol to obtain fragments of 200 nt. T4 PNK (New England Biolabs) treatment was performed to convert 5′-OH to 5′-P and 3′-P and 2′3′-cyclic P (2′3′-cyclic phosphate) to 3′-OH and prepare RNA for adapter ligation during library preparation. Samples were purified using the Zymo Research Oligo Clean and Concentrator kit. Library preparation was done using the NEBNext Multiplex Small RNA Library Prep Set for Illumina according to the manufacturer’s protocol. BluePippin size selection was done using 2% agarose gel cassette (Sage Science) to select for 100–to 600–base pair (bp) products. Quality control at each step was carried out by both Qubit and fragment analyzer. RNA-seq was performed using Illumina NextSeq High-Output kit 2 × 35–bp paired-end reads at Waksman Genomics Core Facility and the Newark Genomics Center, Rutgers University. The similar procedure was performed for RNA library preparation and sequencing of RNA cleavage products produced by target-activated LshCas13a in vitro. RNA-seq in vitro cleavage products was performed at Skoltech Genomics Core Facility. All samples were present in three biological replicates.

### RNA-seq data analysis

All sequencing data and custom scripts used in the analysis are deposed at the Dryad (https://doi.org/10.5061/dryad.sqv9s4n9w) and the Zenodo (https://doi.org/10.5281/zenodo.10595868). The custom scripts can also be found at GitHub (https://github.com/matveykolesnik/LshCas13a_RNA_cleavage).

Raw RNA-seq reads were filtered by quality with simultaneous adapter removal using trimmomatic v. 0.36 (*35*). The exact parameters of trimmomatic run are available in the raw_data_processing.sh file. Adapter content and quality of reads before and after the processing were assessed using FastQC v. 0.11.9. Processed reads were mapped onto reference sequences (RefSeq: NC_000913.3 supplemented with pC002 and pC008 plasmids for nontargeting samples and NC_000913.3 supplemented with pC003_RFP_spacer and pC008 plasmids for targeting samples), using bowtie2 v. 2.3.4.3 (*36*) producing corresponding SAM files. For the TSS detection, processed reads for nontargeting samples with or without RppH treatment were mapped to the NC_000913.2 sequence. To analyze RNA-seq data for in vitro cleavage experiments, RNA reads were mapped to the NC_000913.3 sequence. The exact parameters of bowtie2 run are available in the read_mapping.sh file. Next, for each nucleotide position of each strand of reference sequences, the number of 5′ ends of aligned fragments was counted, producing corresponding tables (see return_fragment_coords_table.py file for details). The obtained tables were joined using merge_ends_count_tables.py script. The differences between the numbers of mapped 5′ ends in targeting and nontargeting samples were analyzed using edgeR package v. 3.26.3 (*37*). The features (here, strand-specific nucleotide positions) containing less than 10 counts per million (CPM) of mapped 5′ ends in less than three targeting samples replicates were excluded from the analysis. The trimmed mean of *M*-value normalization method implemented in edgeR was applied. Next, the edgeR likelihood ratio test was performed. The obtained *P* values were corrected using Benjamini-Hochberg method, and the result tables containing analyzed features with assigned log_2_ fold change (log_2_FC) and adjusted *P* values were written to separate files (see TCS_calling.R file for details). Further, the features were sorted by adjusted *P* values in ascending order, and top 100 features with log_2_FC > 4 (if exist) were considered as putative RNA cleavage sites. To build weblogo plots, the identified RNA cleavage sites were sorted by adjusted *P* values in ascending order, and top 100 features with log_2_FC > 4 were selected for the analysis. Ten nucleotides surrounding the selected sites were obtained from the reference sequences using Biopython toolkit (*38*), and the Logomaker (*39*) module was used to create weblogo plots (see TCS_LRT_weblogo.ipynb file for details). Secondary structures of transcripts were predicted using the RNAfold tool from the ViennaRNA package (*40*) and visualized using the forgi module (*41*) (see draw_hairpin_structures.ipynb file for details). To visualize the cleavage of different tRNAs, the sequences of tRNA genes that were included in the analysis were extracted from annotated NC_000913.3 assembly and aligned using MAFFT v. 7.453 with --maxiterate 1000 --localpair parameters. Each position of the alignment was assigned with the corresponding −log_10_ (adjusted *P* value) depicting the statistical significance of the enrichment of 5′ ends counts in targeting samples over nontargeting samples in Δ10 strain. The positions corresponding to gaps or the positions excluded from the analysis were assigned with zero values. The resulting table was visualized as the heatmap where the intensity of color depicts the −log_10_ (adjusted *P* values). To validate the approach used for identification of RNA cleavage sites, we apply this method to define TSS. The differences between the numbers of mapped 5′ ends in the samples with and without RppH treatment were analyzed in the same way as it was done for the detection of RNA cleavage sites. Predicted TSSs were compared with the *E. coli* MG1655 TSS list from RegulonDB (*42*).

### Volcano plots and Venn diagram construction

To elicit the influence of RNA cleavage on cellular physiology, the analyzed features were assigned to the functional genomic loci. The coordinates (reference ID and strand-specific nucleotide position) of each feature were matched with the coordinates of genomic loci described in the annotations of NC_000913.3 and pC008 (*2*) sequences. NC_000913.3 annotation was retrieved from National Center for Biotechnology Information (NCBI) GenBank. Custom script merge_TCS_with_genome_annotation.R was used for this purpose. For further analysis, the features overlapping with annotated coding sequences (CDS; i.e., open reading frame), tRNA, and rRNA genes were selected. The results of the likelihood ratio test were visualized using the volcano plot approach in a space of log_2_FC and −log_10_ (adjusted *P* value) dimensions; thus, each feature was put on a plot at the position corresponding to the calculated log_2_FC and −log_10_ (adjusted *P* value). The dots were colored according to the types of assigned genomic loci (rRNA, CDS, or tRNA). To visualize the thresholds (top 100 features with log_2_FC > 4), the dashed horizontal and vertical lines were added to the plots. To compare the patterns of RNA cleavage across the experiments, the sets of predicted RNA cleavage sites (i.e., the strand-specific nucleotide positions) from different experiments were intersected. The results of the intersection were depicted by Venn plot. Because, in different experiments, the same RNAs could be cleaved in different positions, the sets of transcripts were intersected. Because, in *E. coli*, few tRNAs are encoded by more than one gene, tRNA genes with the same nucleotide sequences were collapsed (e.g., glnU and glnW were collapsed to glnUW). If RNA cleavage site was predicted in at least one tRNA genes belonging to the group of identical tRNAs, then the cleavage was attributed to the collapsed tRNA.

### RNA coverage analysis

The coverage of reference sequences by RNA reads per nucleotide position of each strand was calculated using a custom script (return_fragments_coverage.py). CPM of mapped read normalization was applied to the obtained values. To visualize the coverage, the mean of CPM values was plotted across the nucleotide positions; the SEM was depicted by whiskers. To visualize the coverage by mapped 5′ ends, the same normalization and visualization approaches were applied to the 5′ end counts.

### Differential expression

After the mapping of RNA-seq reads on the reference sequences, the numbers of mapped RNA fragments per annotated genomic loci were calculated using featureCounts function from Rsubread (*43*) package in R (*see* build_featureCounts_tables.R script). Differential gene expression analysis was conducted using DESeq2 (*44*) package in R.

### Analysis of tRNA anticodon loops

To investigate the potential cleavage of tRNAs in *L. shahii* and *Homo sapiens* by the activated LshCas13a enzyme, we compare the sequences of *E. coli* tRNAs [tRNA^lys^ (UUU), tRNA^glu^ (UUC), tRNA^gln^ (UUG), and tRNA^thr^ (UGU)] that were demonstrated to undergo cleavage in the experiments and corresponding tRNAs from *L. shahii* (RefSeq NZ_AP019827.1) and *H. sapiens* (http://gtrnadb.ucsc.edu/Hsapi19/Hsapi19-seq.html) genomes. We specifically extracted the sequences corresponding to the anticodon stem loops and visualized these tRNA structures using the forgi module (*41*) and subsequently generated a weblogo plot using the logomaker package.

### Analysis of the relative level of aminoacylated tRNAs

To measure the relative amount of charged tRNAs, we developed LC-MS–based method for the GATRAL. Under GATRAL, tRNA extracted from the cells is subjected to the acetic anhydride treatment to protect the aminoacyl moiety of charged tRNA followed by RNase digestion. The resulting aminoacylated adenosine signal quantified by LC-MS reflects the quantity of the corresponding aminoacylated tRNA. To isolate the tRNA-enriched fraction, cell pellets from 10-ml cultures were resuspended in 0.5 ml of 300 mM Na-acetate buffer (pH 4.5)/10 mM EDTA and mixed with 0.5 ml of phenol saturated with 0.1 M citrate buffer (pH 4.3). Extraction was carried out at 4°C with gentle rotation for 15 min. After phase separation, the aqueous phase was collected, and the phenol extraction step was repeated. The resulting RNA was precipitated with 0.5 ml of isopropanol; the precipitate was washed with 100 mM Na-acetate buffer (pH 5) in 70% ethanol and air-dried. tRNA (10 μg) was dissolved in 60 μl of 100 mM Na-acetate buffer (pH 5.0), followed by the addition of 1 μl of acetic anhydride. The reaction was incubated on ice for 1 hour and then supplemented with an additional 1 μl of acetic anhydride, followed by 1 hour of incubation on ice. Upon reaction completion, the concentration of Na-acetate was adjusted to 300 mM and reaction volume was adjusted to 250 μl. The acetylated tRNA was precipitated by the addition of 1 volume of isopropanol. Aminoacyl-tRNA (10 μg) modified with acetyl group was digested with 100 U of RNase I (Ambion, USA) and 1000 U of RNase T1 (Thermo Fisher Scientific, USA) in 30 mM tris-HCl (pH 7.0) and 50 mM NaCl at 37°C for 30 min. LC-MS analysis was performed using Agilent 1200 HPLC equipped with the UV and 6550 iFunnel QTOF LC/MS detectors and Jet Stream Technology ion source (Agilent, USA). The products of RNase I digestion were separated by Poroshell 120 SB-C18 column (2.7 m, 2.1 mm by 100 mm) (Agilent, USA) at 40°C using a linear (0 to 80%) gradient of acetonitrile in 5 mM ammonium acetate buffer (pH 5.2) at 0.2 ml/min. The electrospray source was set to positive ion mode at 4 kV, 290°C. Data acquisition was performed in the mass/charge ratio (*m/z*) range of 200 to 1100 at the rate of two spectra/s. Fragmentation spectra were recorded in AutoMSMS mode. Data were analyzed using MassHunter Qualitative Analysis 10.00 software (Agilent, USA) with Quartic/Quintic Savitzky-Golay smoothing function (function width, 30 points) and ChemStation integrator selector (default options, but advanced baseline correction mode). For normalizing the signals of aminoacylated adenosines between different samples, we used [^13^C]-GMP (*m/z*, 365) signal (from digested tRNA). Signals from each aminoacylated adenosine were MS/MS confirmed. To validate GATRAL, *E. coli* BW25113 cells were treated with 30 μM McC at 37°C for 20 min. Untreated cells were used as a control. tRNA was purified and subjected for GATRAL assay.

### Statistical analysis

The statistical methods and tests used for each analysis are specified in Materials and Methods.
