# Supplementary material for: tRNA anticodon cleavage by target-activated CRISPR-Cas13a effector
Source: Sci Adv. 2024 Apr 24;10(17):eadl0164. doi: 10.1126/sciadv.adl0164 (PMC11042736; doi:10.1126/sciadv.adl0164)
Supplement: Supplementary file 1 — Supplementary Text Figs. S1 to S17 Tables S1 to S6 [file sciadv.adl0164_sm.pdf]

Science Advances
AAAS

Supplementary Materials for

tRNA anticodon cleavage by target-activated CRISPR-Cas13a effector

Ishita Jain *et al.*

Corresponding author: Ekaterina Semenova, semenova@waksman.rutgers.edu;
Konstantin Severinov, severik@waksman.rutgers.edu

Sci. Adv. **10**, eadl0164 (2024)
DOI: 10.1126/sciadv.adl0164

This PDF file includes:

Supplementary Text
Figs. S1 to S17
Tables S1 to S6

Supplementary Text

Transcription start sites in targeting RNA samples

Since the supposed collateral RNA degradation could cause global changes in *E. coli* transcriptome, we should consider a possibility that some of the observed enrichments in 5' end counts between targeting and nontargeting samples could correspond to transcription start sites (TSSs) that are presented in targeting but not in nontargeting samples. To test this hypothesis, we performed dRNA-seq of total RNA samples extracted from targeting and nontargeting cell cultures. The prediction of TSSs was performed in the same way as it was done for the RNA cleavage sites. To verify the method of TSS detection, the set of top-1000 (selected after sorting by adjusted p-value in ascending order) of TSSs predicted in nontargeting samples was compared with the *E. coli* MG1655 TSSs list from RegulonDB. Specifically, the number of predicted TSSs that coincide with the TSSs (in case of single position TSSs) or fall into TSSs clusters (in case of cluster TSSs) described in RegulonDB was calculated. From the top-1000 of the predicted TSS 306 are presented in RegulonDB. Given that the total number of nucleotide positions on both strands in NC_000913.3 is 9279350, and the number of TSSs in RegulonDB (counting all nucleotide positions in cluster TSSs) is 6799, it is highly improbable (p-value ~0, Fisher exact test) to obtain 306 TSSs by random sampling, indicating that the approach of dRNA-Seq analysis is valid. Next, the sets of top-1000 of TSSs predicted in targeting cells and top-1000 of predicted RNA cleavage sites were overlapped. There were only five sites shared between those two sets, suggesting that the emergence of additional TSSs has a little contribution to the observed 5' ends enrichment between targeting and nontargeting samples.

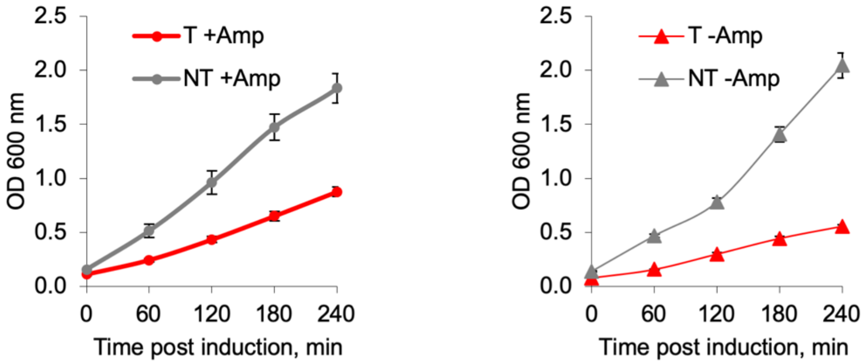

Fig. S1. Growth retardation does not depend on the presence of an antibiotic essential for the selective maintenance of the plasmid encoding the target RNA. The growth of targeting and nontargeting cells in media with (left) and without (right) ampicillin to selectively maintain the target plasmid.

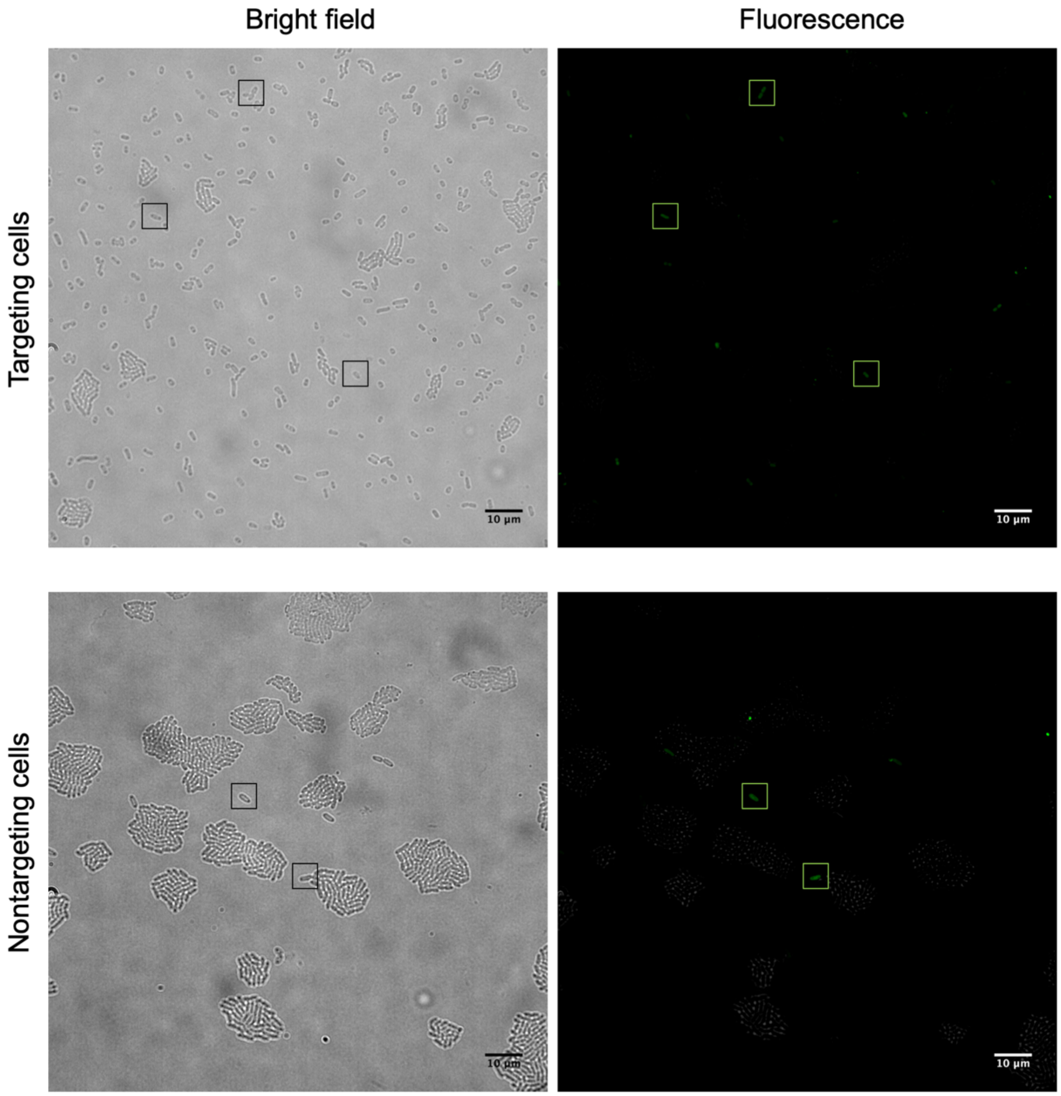

Fig. S2. YOYO-1 staining revealed a small number of dead cells equally in targeting and nontargeting cultures. Representative bright-field and fluorescence images of targeting and nontargeting cultures stained with a membrane-impermeable dye YOYO-1 for the detection of dead cells. Bright-field and fluorescence images were acquired for the same field of view at 168 minutes post-induction. Small boxes indicate dead cells fluorescing green due to YOYO-1 bound to DNA. Fraction of dead cells was 0.81 ± 0.61 % in targeting cultures and 1.18 ± 0.39 % in nontargeting cultures (mean ± SEM). Statistics for dead cells' fraction was calculated from three biological replicates for targeting and nontargeting cultures. At least 100 cells were analyzed for each experiment.

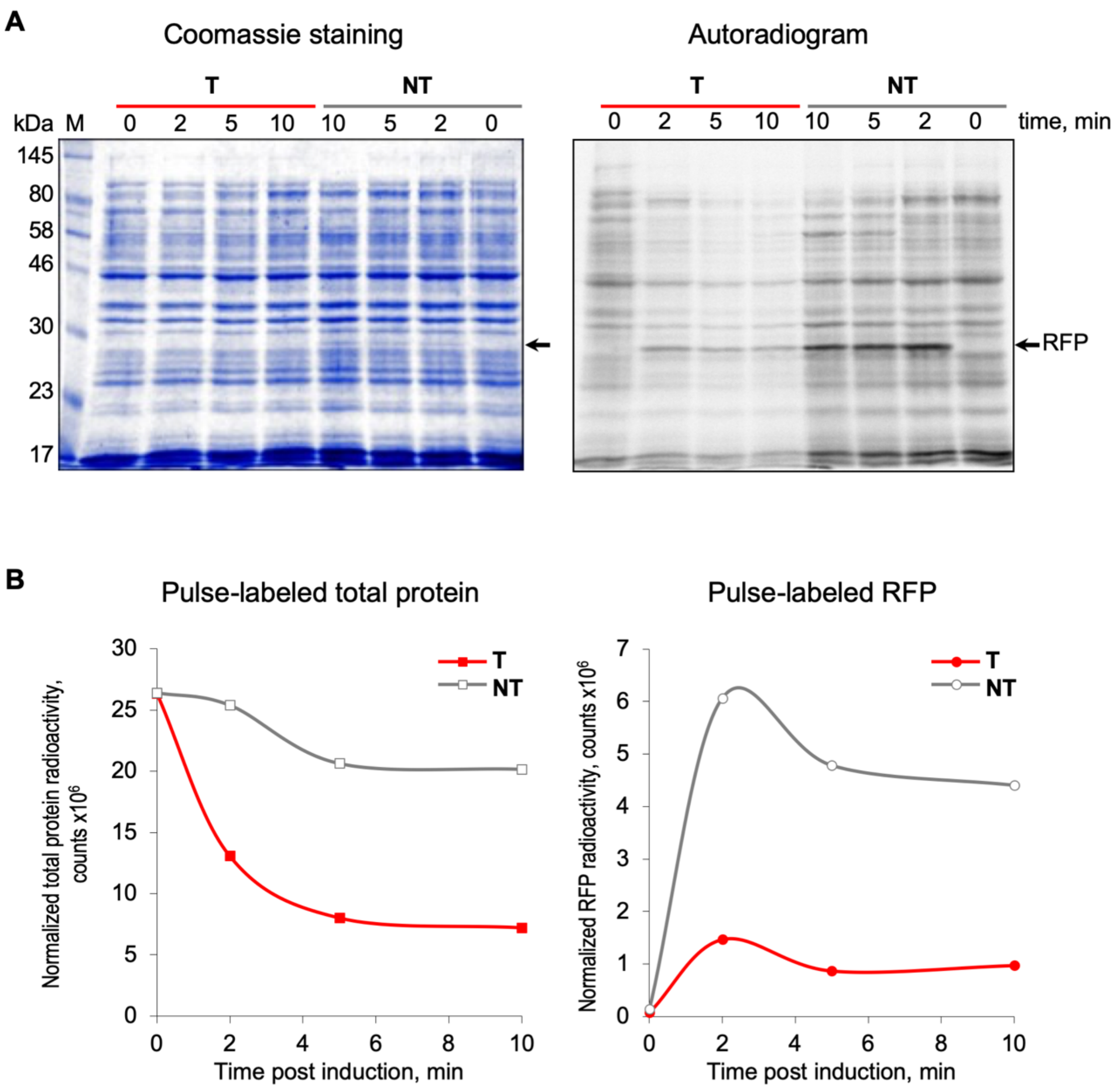

Fig. S3. Time course of protein synthesis in targeting (T) and nontargeting (NT) cells after RFP induction. (**A**) Protein samples obtained during pulse-labeling of *E. coli* cells with [^{35}S]-methionine, were separated by MOPS/SDS 10% PAGE, gels were stained with Coomassie, and quantified using a Phosphorimager. The position of RFP on the gel and the autoradiogram is indicated by a black arrow. (**B**) Graphs plots show the [^{35}S]-radioactivity of pulse-labeled RFP and total cellular proteins in targeting (T, red) and nontargeting cells (NT, grey).

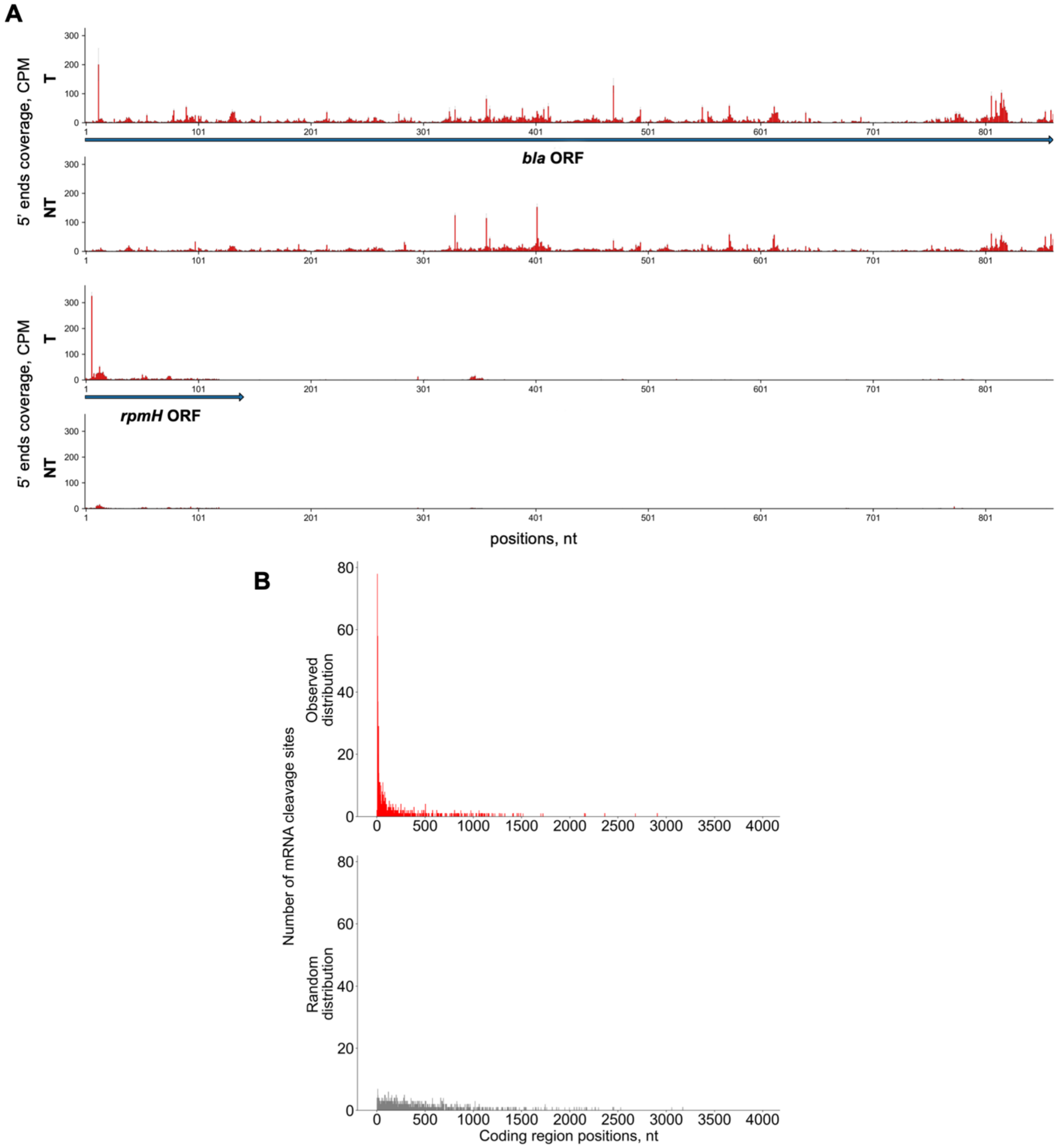

Fig. S4. mRNA cleavages at the beginning of coding sequences in targeting cells. (**A**) CPM (counts per million) normalized coverage of 5' ends of transcripts mapped to the coding regions of *bla* (top panel) and *rpmH* (bottom panel, shown in the same scale for simplicity) genes in targeting (T) and nontargeting (NT) samples of *E. coli* cells. The horizontal axes depict the nucleotide positions of the coding regions. Red bars correspond to mean (± SEM) CPM normalized counts of mapped 5' ends from three biological replicates. (**B**) The distribution of the top-1000 of mRNA cleavage sites (sorted by adjusted p-values in ascending order) across the positions of the coding regions revealed efficient cleavage at the beginning of the coding sequences.

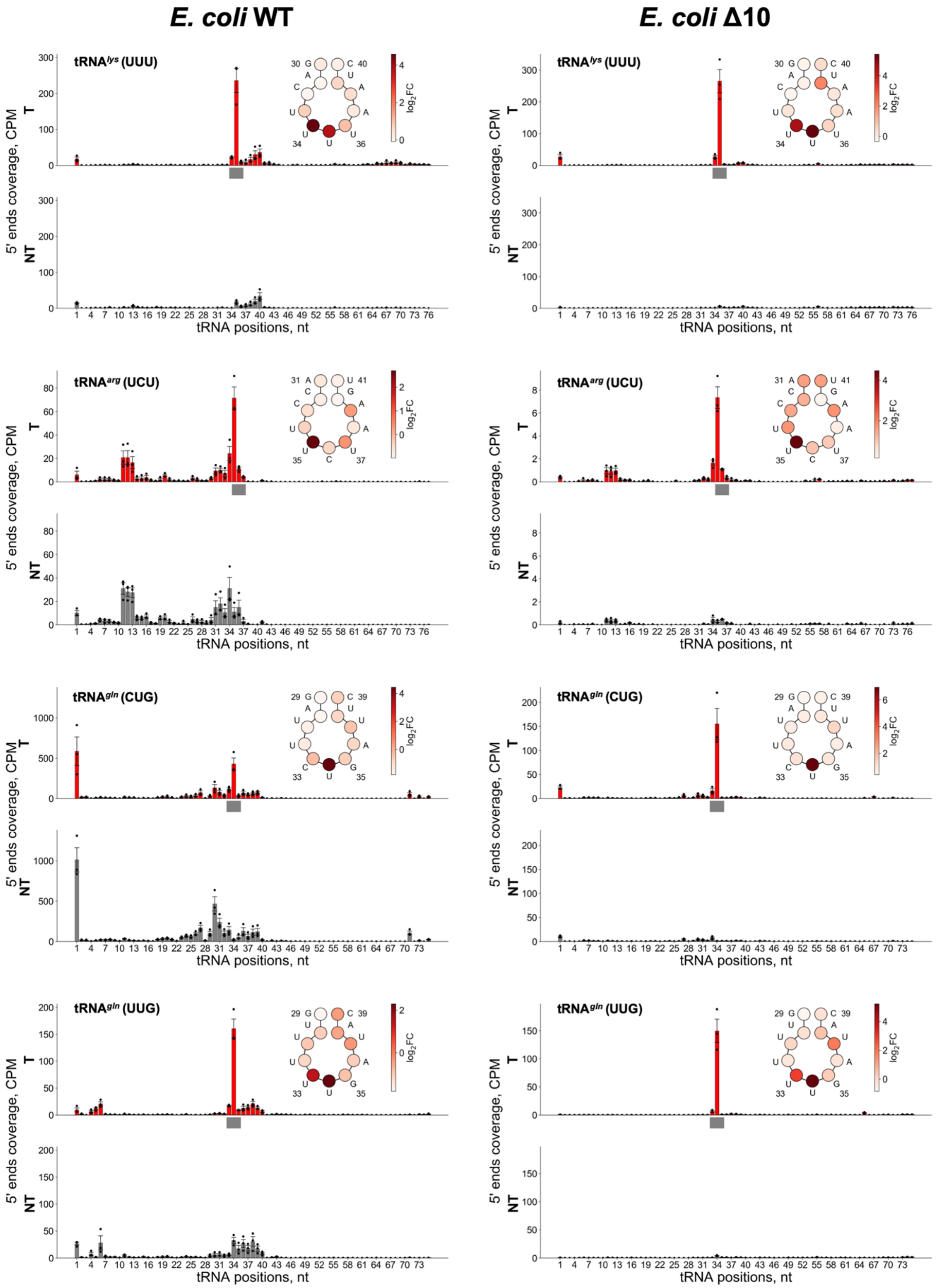
E. coli WT
E. coli Δ10
tRNAlys (UUU)
tRNAarg (UCU)
tRNAgln (CUG)
tRNAgln (UUG)
5' ends coverage, CPM
T
NT
tRNA positions, nt
log2FC

Fig. S5.

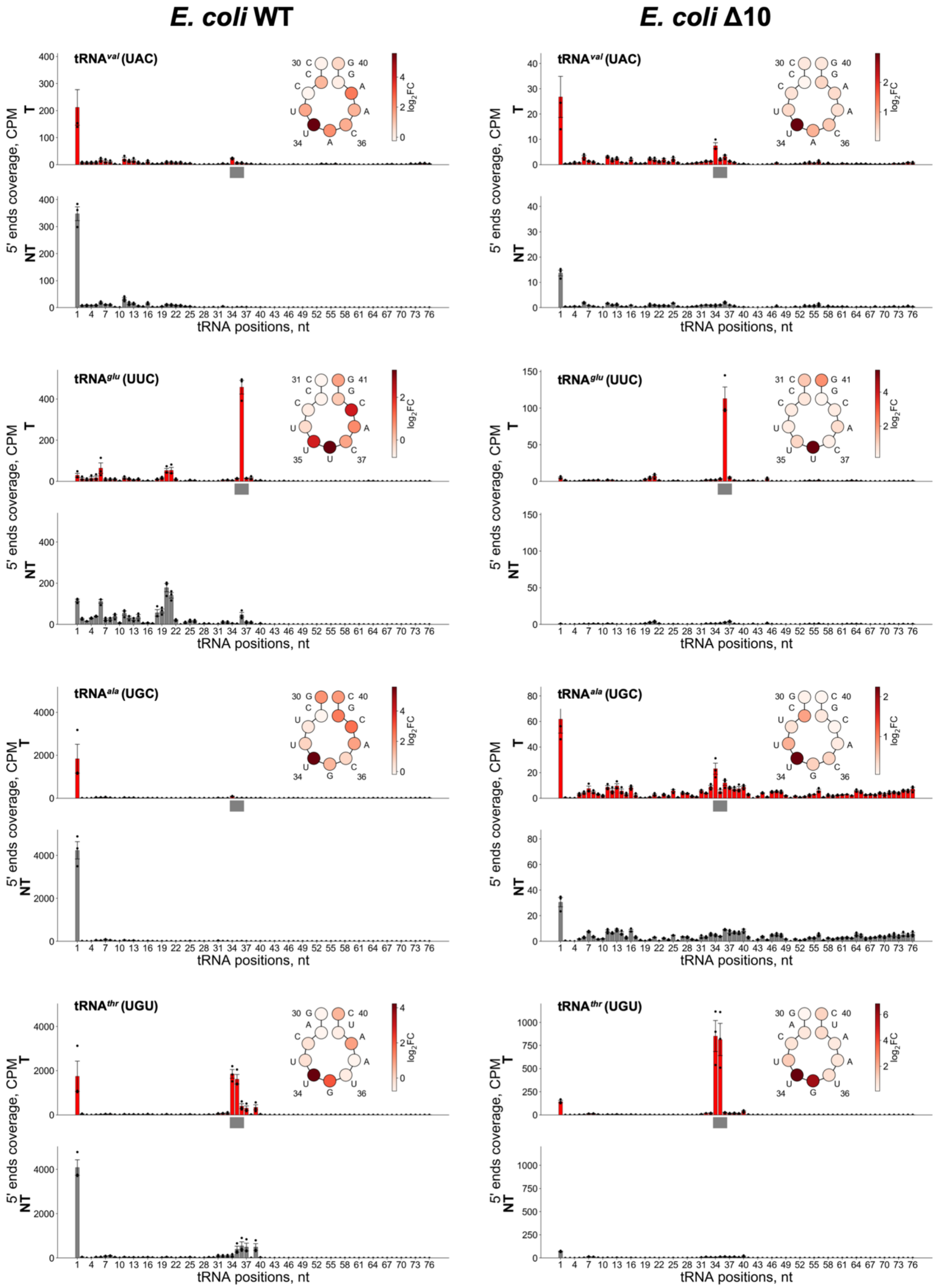

Fig. S5 continued. Visualization of cleavage sites in tRNAs in wild-type and Δ10 *E. coli* cells. The analysis was performed in the same way as in Fig. 2, A and B: CPM normalized coverage of 5’ ends of transcripts mapped onto tRNA genes in targeting (T, red bars) and

nontargeting (NT, grey bars) samples of *E. coli* cells (mean ± SEM from three biological replicates). The horizontal axis depicts the nucleotide position of tRNA. The region corresponding to the anticodon is shown by a grey bar. At the top right part of each panel the scheme of the tRNA anticodon loop is depicted; circles correspond to tRNA nucleotides. Each circle is colored according to color scheme where the intensity of color corresponds to values of log_2FC between mean 5' ends CPMs in targeting and nontargeting samples.

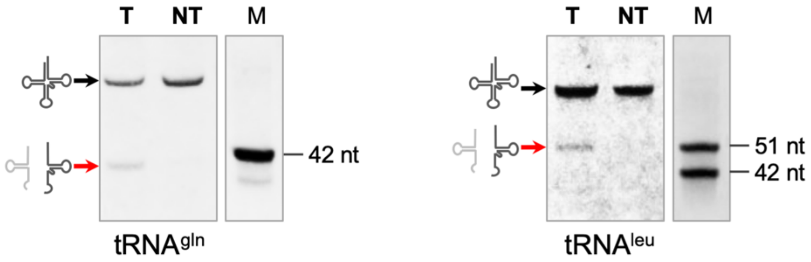

Fig. S6. tRNA^{gln} and tRNA^{leu} cleavage. tRNA^{gln} (left) and tRNA^{leu} (right) cleavage in targeting cells was quantified using Northern Blot hybridization with a 3'-fluorescein-labeled probe complementary to tRNA. RNA samples from targeting (T) and nontargeting cells (NT) were analyzed. Black arrows depict the position of full-size RNA, red arrows depict the position of cleavage product. The gel images are representative of three independent experiments. The extent of cleavage determined for three biological replicates of targeting samples is 9.2 ± 2.4 % for tRNA^{gln} and 10.6 ± 0.9 % for tRNA^{leu} (mean ± SEM, n=3).

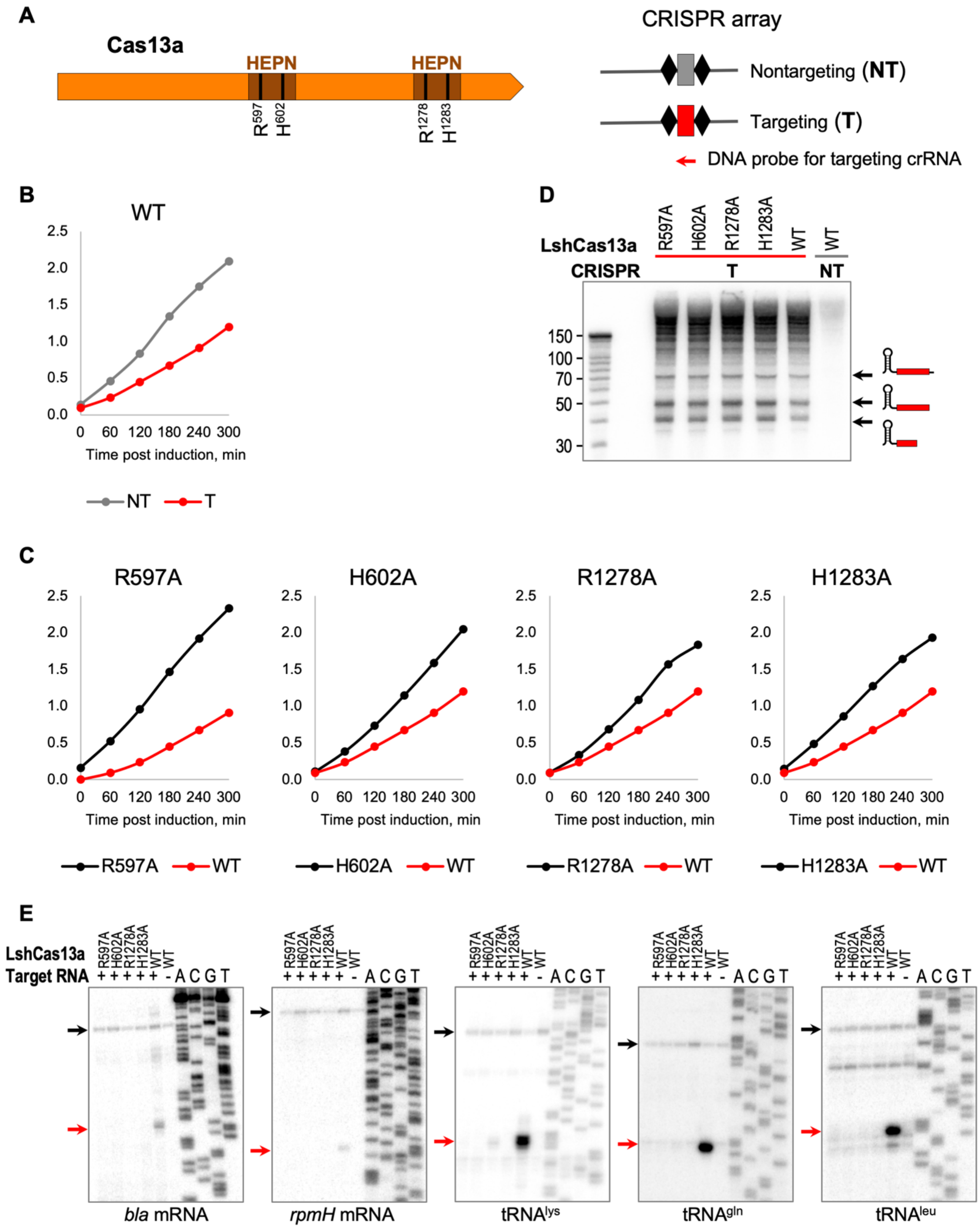

Fig. S7. Effect of HEPN catalytic residue mutations on cell growth, crRNA processing, and RNA cleavage in cells expressing target-activated Cas13a. (**A**) Schematic of the domain organization of LshCas13a protein showing the catalytic residues of HEPN domains. CRISPR array structures for cells used in these experiments are shown on the right. (**B**), (**C**) A single

substitution of any of four catalytic residues restores growth in cells expressing mutant Cas13a, crRNA and complementary target RNA, the growth rate is similar to that of nontargeting control cells expressing wild type Cas13. (**D**) HEPN mutations do not affect crRNA processing. Northern Blot hybridization with a probe complementary to the crRNA1 spacer targeting RFP mRNA. (**E**) HEPN mutations abolish mRNA and tRNA cleavages.

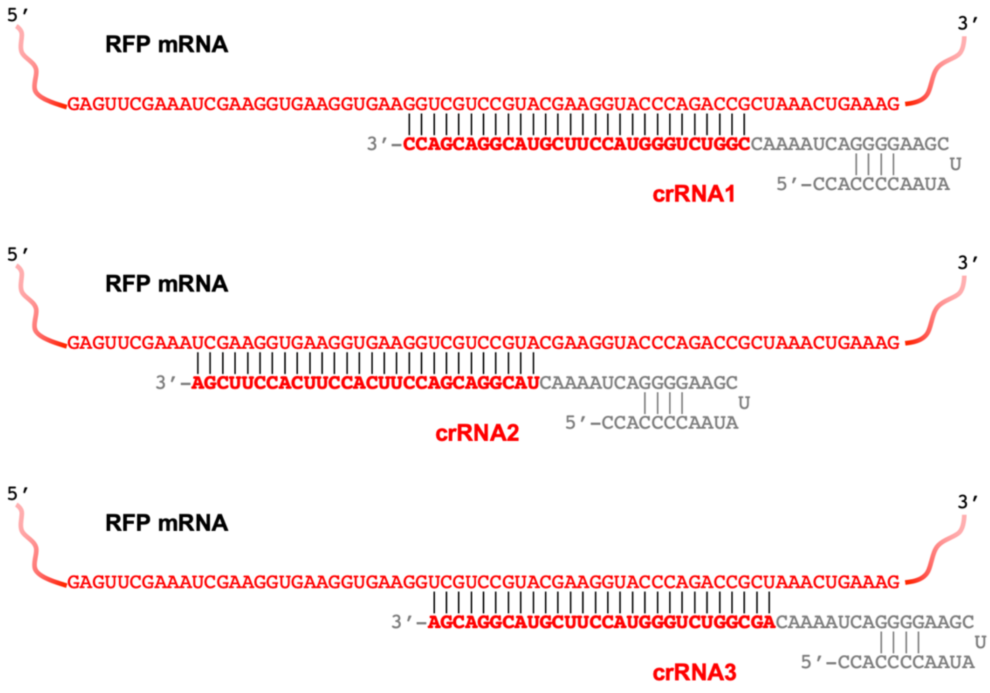

Fig. S8. crRNAs targeting RFP mRNA. Schematic depicts crRNA structures targeting three different positions in RFP mRNA (shown in red) with crRNA spacer sequence shown in red and repeat sequence shown in grey.

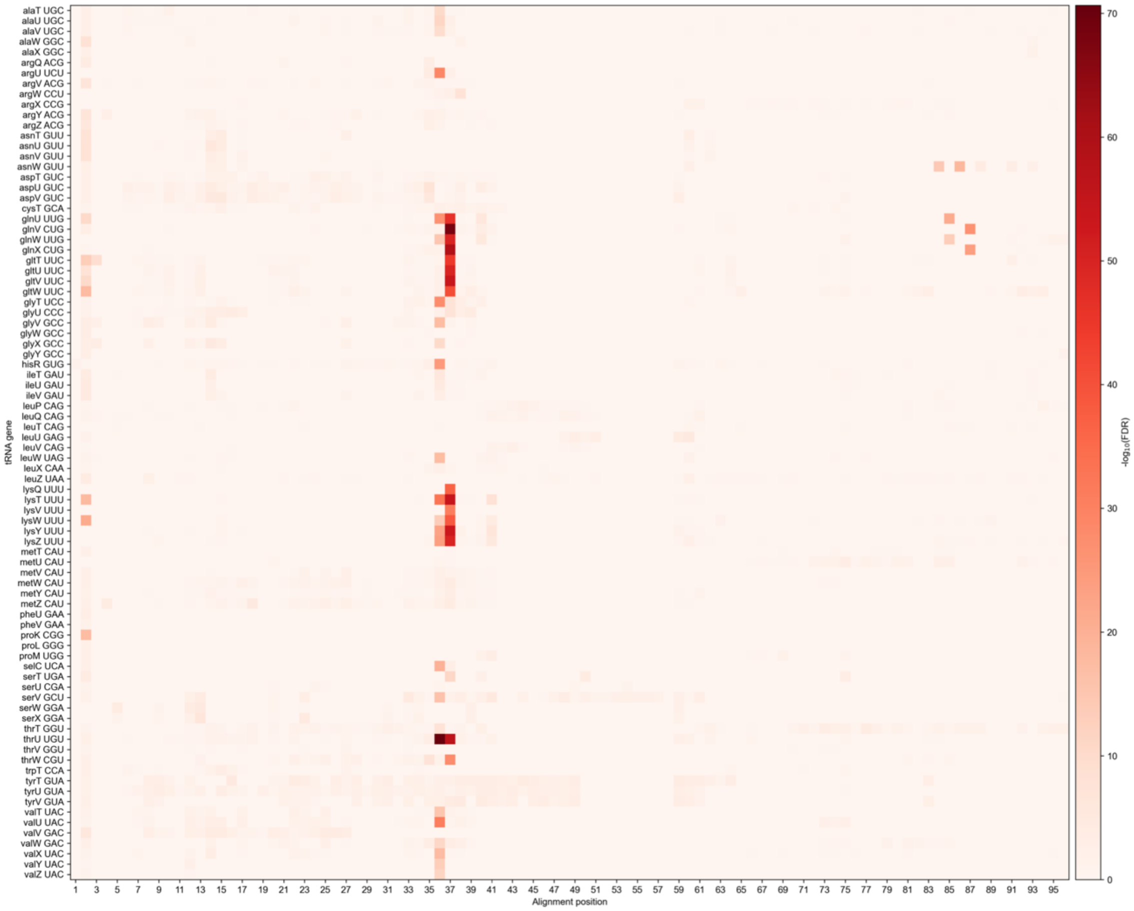

Fig. S9. The preference of the cleavage of different tRNAs in Δ10 strain. The nucleotide sequences of tRNA genes were aligned. The horizontal axis depicts the position of the multiple sequence alignment. The vertical axis corresponds to different tRNA genes. The positions of each tRNA in the alignment were assigned with the corresponding -log10 (adjusted p-value) that represents the statistical significance of the cleavage at the given position. The positions corresponding to gaps or the positions excluded from the analysis were assigned with zero values. The resulting table was visualized as the heatmap where the intensity of color depicts the -log10(adjusted p-value).

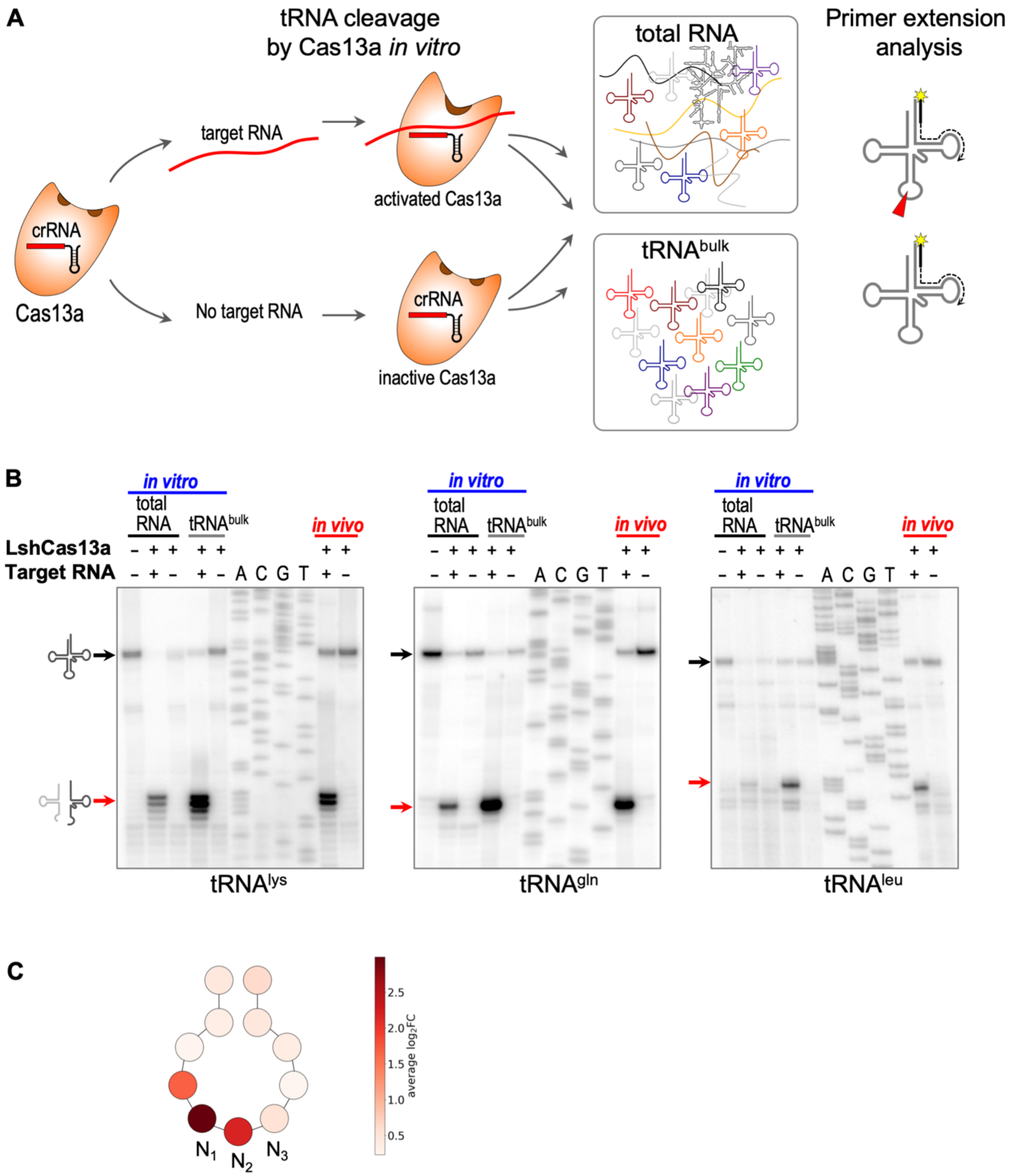

Fig. S10. tRNA cleavage *in vitro*. (**A**) Schematic presentation of *in vitro* cleavage assay: target-activated Cas13a or inactive control are supplemented with total RNA or bulk tRNA for cleavage. Cleavage products analyzed by primer extension assay using a radio-labeled primer complementary to the 3'-end of specific tRNA. (**B**) Primer extension assay revealed that the position of cleavage sites at the tRNA anticodon loop is unchanged when total *E. coli* RNA or bulk *E. coli* tRNA is used as cleavage substrate *in vitro* and matches those observed *in vivo*. (**C**) Schematic depiction of the tRNA anticodon loop; circles correspond to tRNA nucleotides. Each circle is colored according to color scheme where the intensity of color corresponds to average

value of log_2FC between average 5’ ends CPMs in targeting and nontargeting samples across all tRNA genes detected in the *E. coli* genome.

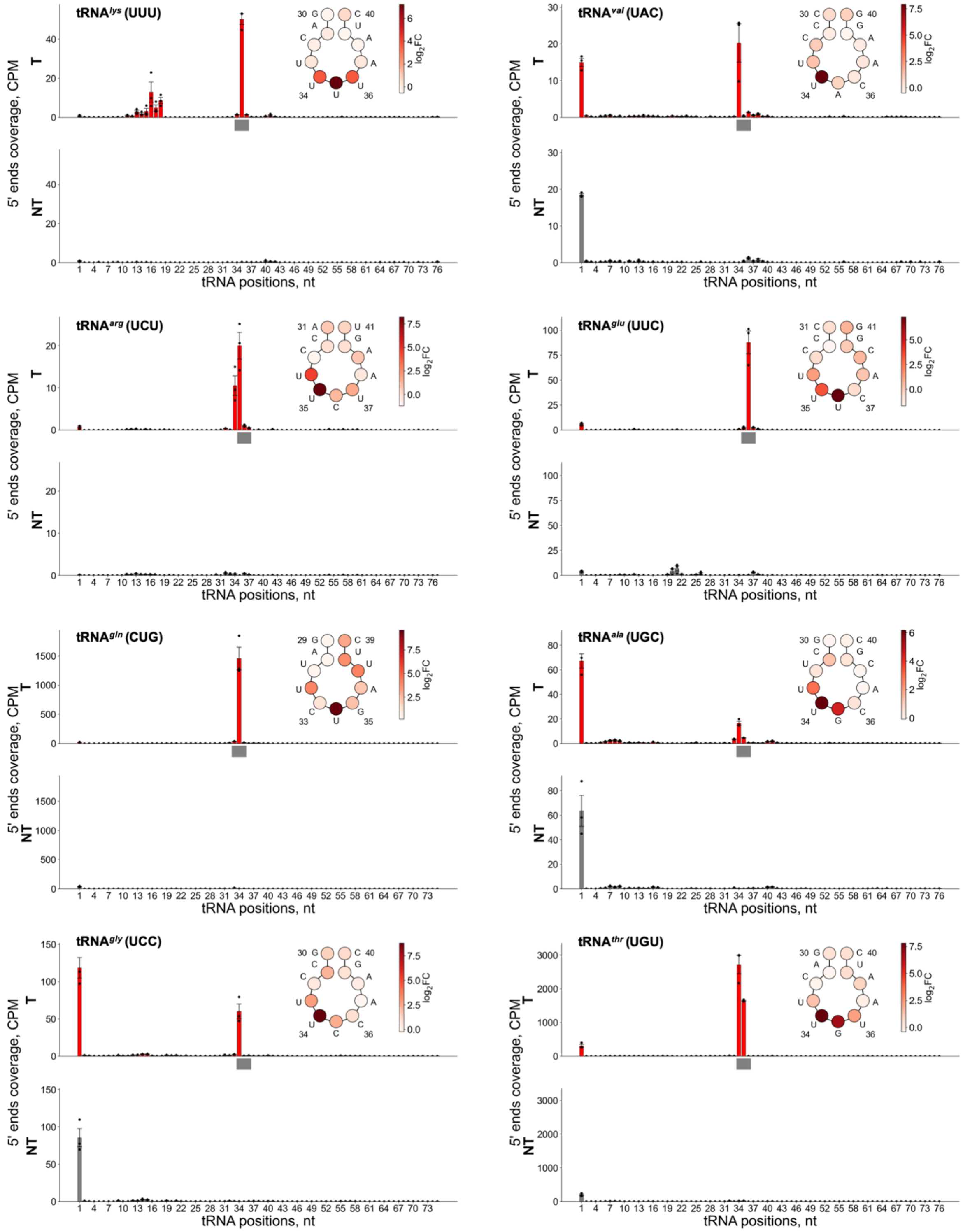

Fig. S11. Mapping *in vitro* tRNA cleavage sites. CPM normalized coverage of 5’ ends of transcripts mapped onto tRNA genes in targeting (T, red bars) and nontargeting (NT, grey bars) *in vitro* samples (mean ± SEM from three independent experiments).

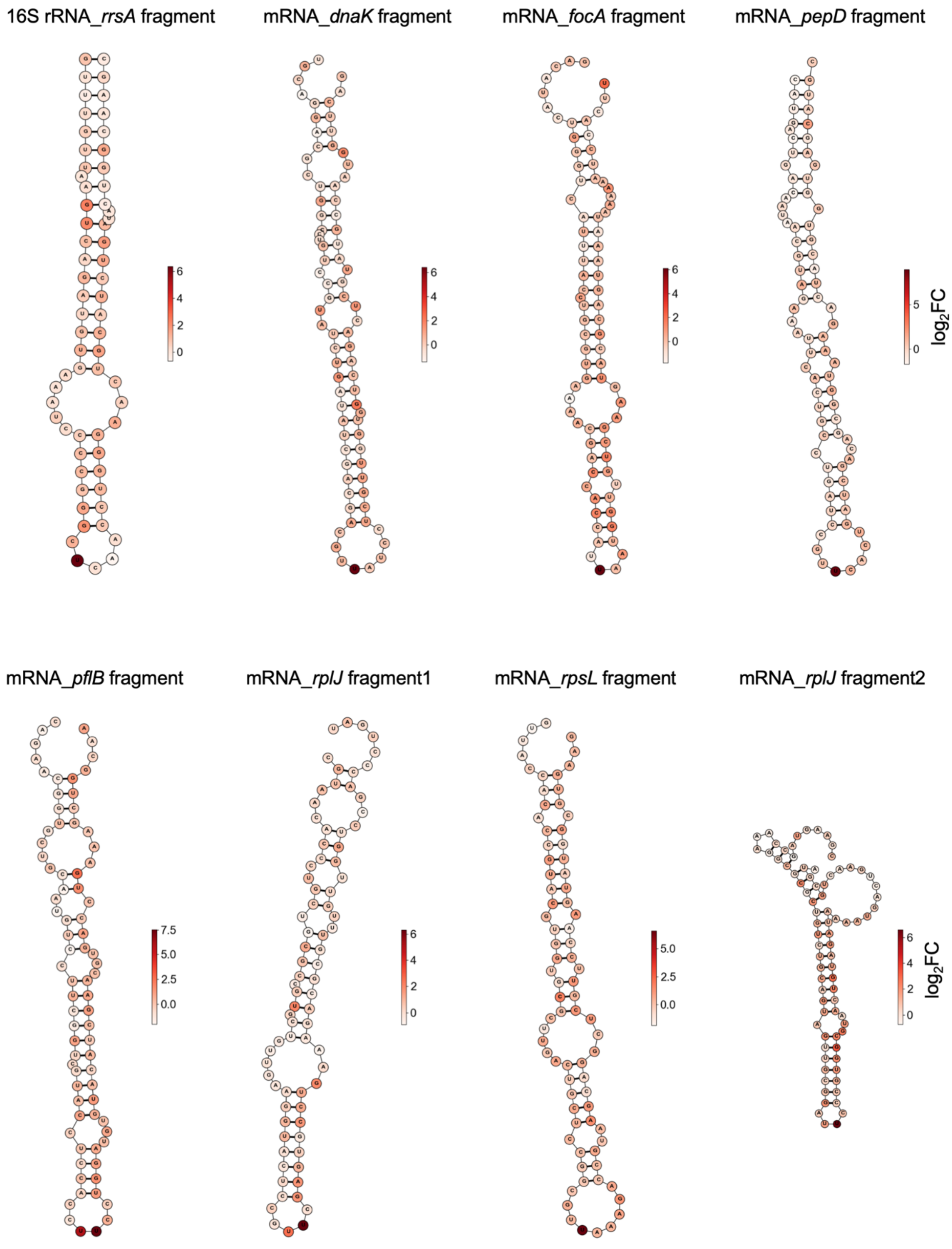

Fig. S12. Non-tRNA cleavages by target-activated Cas13a *in vitro*. Mapping of several *in vitro* cleavage sites detected in non-tRNA substrates on the predicted secondary structures of RNA molecules: eighty nucleotides surrounding the analyzed cleavage sites were extracted, and the RNA secondary structures were predicted using RNAfold program (see Methods). Each circle is

colored according to color scheme where the intensity of color corresponds to values of log_2FC between average 5’ ends CPMs in targeting and nontargeting samples. Non-tRNA cleavages at uridine residues located at small loops mimic tRNA cleavages at *in vitro* conditions.

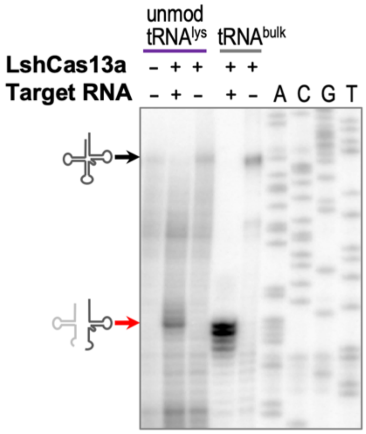

Fig. S13. LshCas13a cleaves synthetic tRNA^{lys} at the anticodon loop. Nucleoside modifications in tRNA are not required for cleavage.

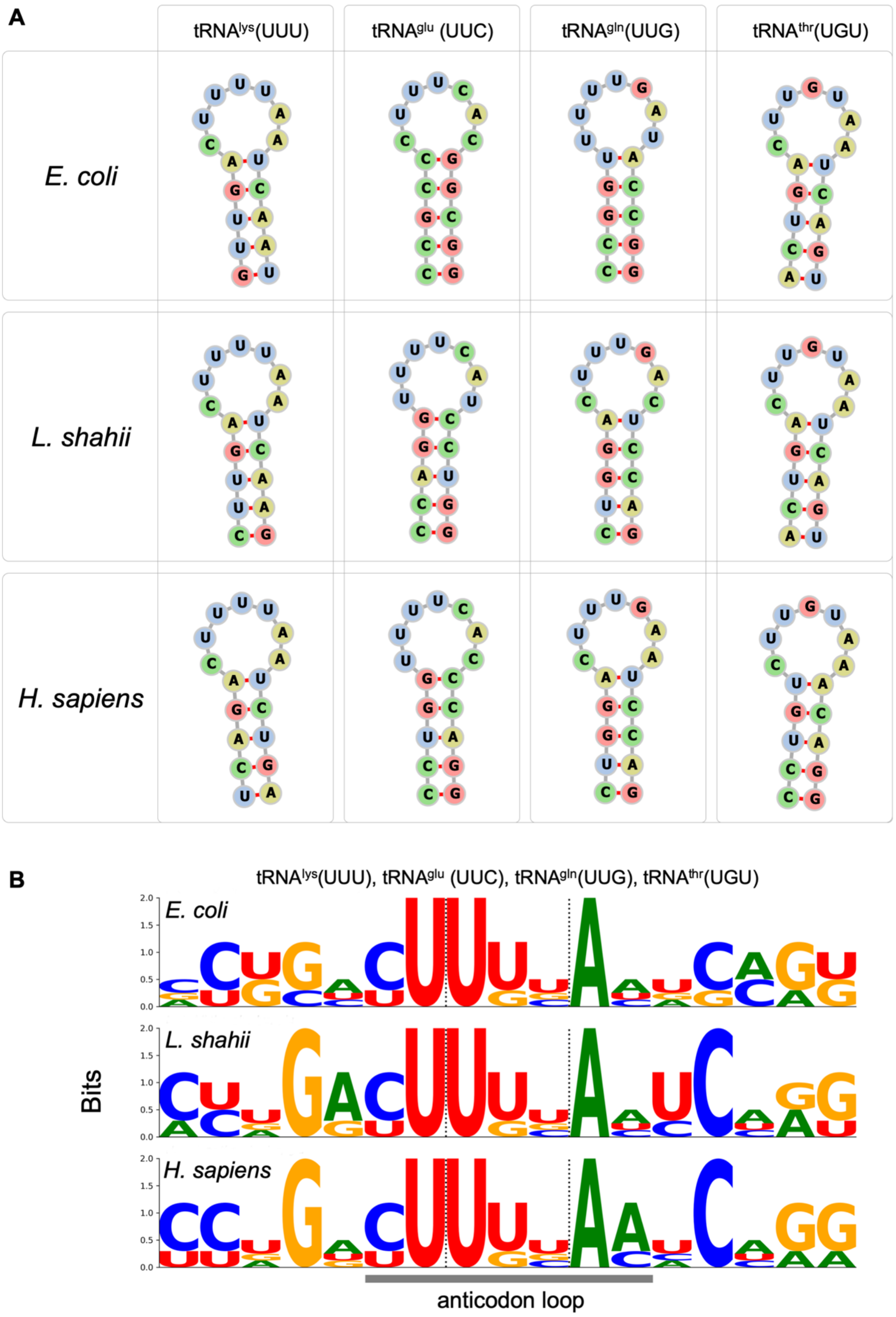

Fig. S14. The tRNA anticodon loop sequences of preferred cleavage substrates for LshCas13a are strongly conserved among *E. coli*, *L. shahii*, and *H. sapiens*. (A) The

anticodon stem-loop structures of $tRNA^{lys}$(UUU), $tRNA^{glu}$(UUC), $tRNA^{gln}$(UUG), and $tRNA^{thr}$(UGU). (**B**) Weblogo plots built for anticodon stem-loop sequences from four tRNAs efficiently cleaved by target-activated LshCas13a. The region corresponding to the anticodon loop is shown by a grey bar, and the position of anticodons is depicted by dash lines.

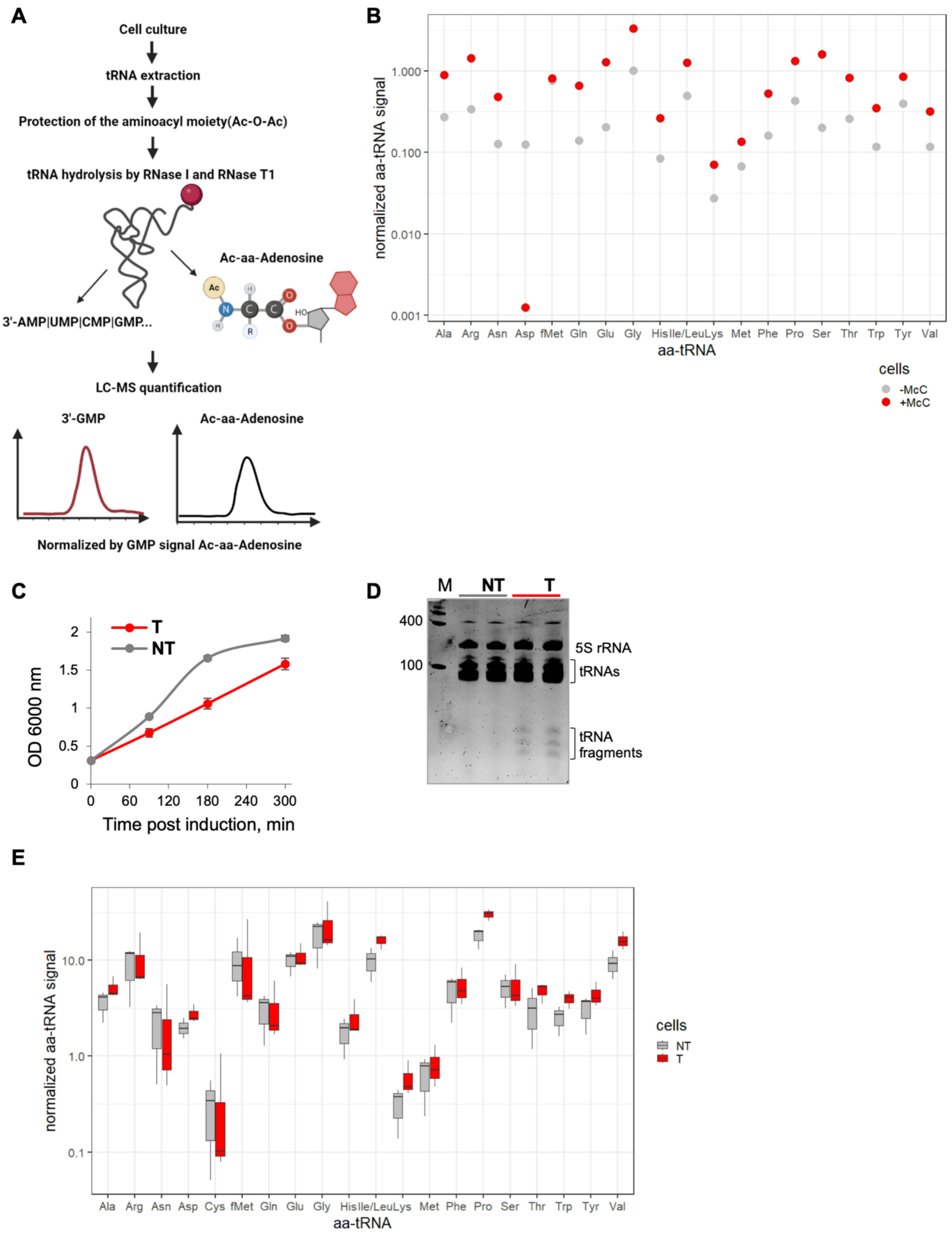

Fig. S15. tRNA cleavage by target-activated Cas13a does not affect aminoacylation level. (A) Schematic presentation of the global analysis of tRNA aminoacylation level (GATRAL) using

LC-MS: tRNA extracted from *E. coli* cells was treated with acetic anhydride (Ac-O-Ac) to protect the aminoacyl moiety of charged tRNA (aa-tRNA) and then hydrolyzed by RNase I and RNase T1 to generate the acetylated derivatives of aminoacylated 3'-terminal adenosine of aa-tRNAs (Ac-aa-Adenosine). The resulting Ac-aa-Adenosine products were quantified using LC-MS. The relative amount of each aa-tRNA was determined as the corresponding Ac-aa-Adenosine signal normalized to the [^{13}C]-GMP signal (*m/z* 365). (**B**) Validation of the LC-MS-based GATRAL assay for quantification of aa-tRNA level using tRNA isolated from *E. coli* cells treated with microcin C (McC), an antibiotic specifically inhibiting aspartyl-tRNA synthetase (*14*). As expected, a dramatic decrease in the relative amount of aspartyl-tRNA was observed in the McC-treated cells (red dots) compared to untreated cells (grey dots). The data from one representative experiment is shown. (**C**) Targeting of RFP mRNA by LshCas13a leads to growth suppression in targeting (T, red) cells; no such effect is observed in nontargeting (NT, grey) control. (**D**) tRNA isolated 15 minutes post-induction from targeting cells (T) contained apparent cleavage products that were not detected in tRNA samples isolated from nontargeting cells (NT) cells. (**E**) Summary of GATRAL results for tRNA samples from cells expressing Cas13a. For ease of visualization, normalized signals for each aa-tRNA showing the results from three biological replicates of targeting (shown in red) and nontargeting (shown in grey) cells are presented as boxplots (median, the middle value in the data set; the boundary of the lower whisker is the minimum value of the data set, and the boundary of the upper whisker is the maximum value of the data set).

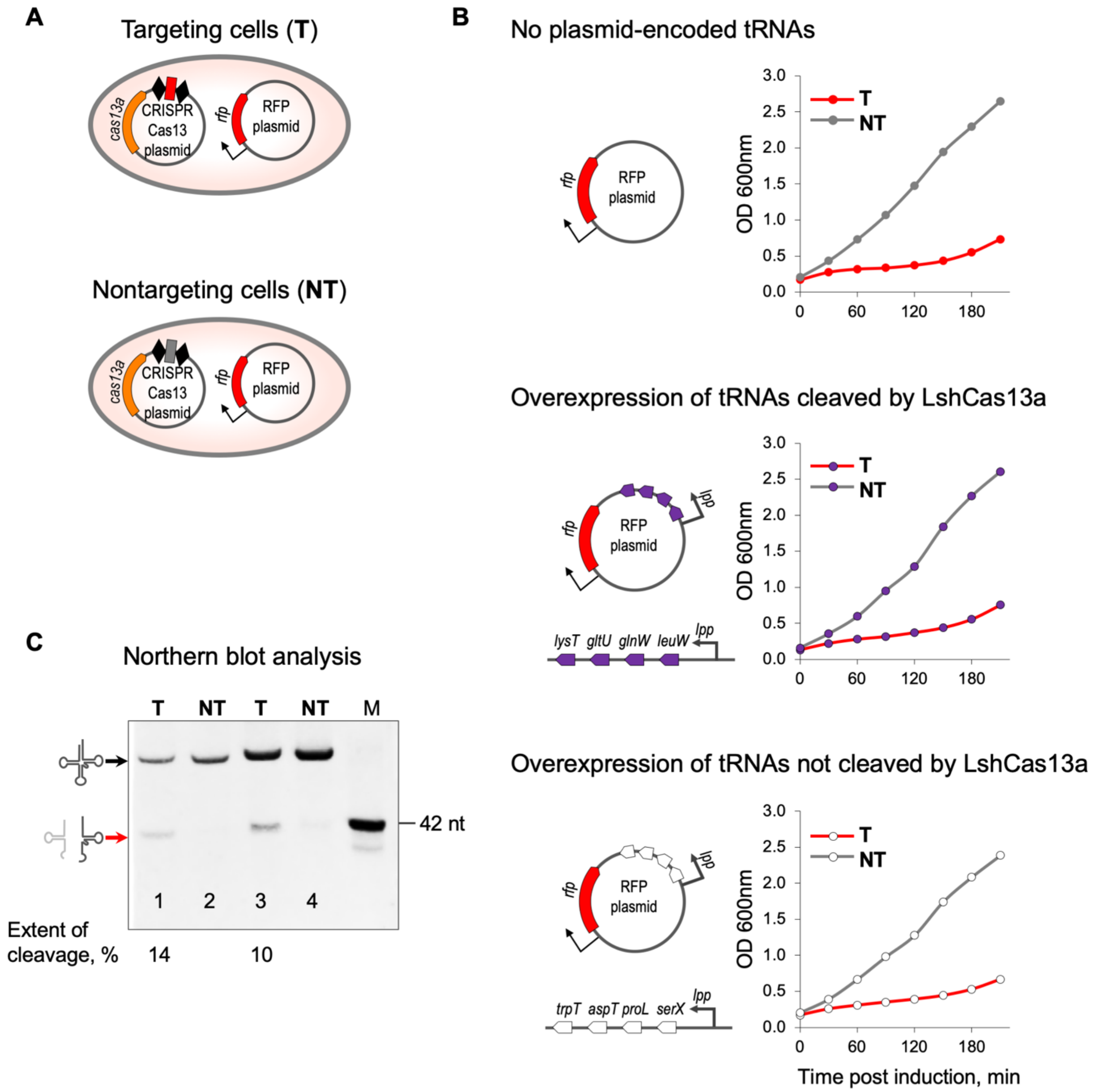

Fig. S16. Expression of additional tRNAs does not rescue cell growth inhibition caused by Cas13a targeting of a non-essential RFP gene transcript. (**A**) A scheme of targeting and nontargeting *E. coli* cells. (**B**) Growth of cultures of targeting and nontargeting cells carrying the plasmids expressing a set of tRNA that are cleaved or not cleaved by target-activated Cas13a, or target plasmid without tRNA genes. (**C**) A representative Northern blot showing the state of $tRNA^{gln}$ (subject to Cas13 target-activated cleavage) in cells expressing (lanes 3 and 4) or not expressing (lanes 1 and 2) additional $tRNA^{gln}$ from a plasmid.

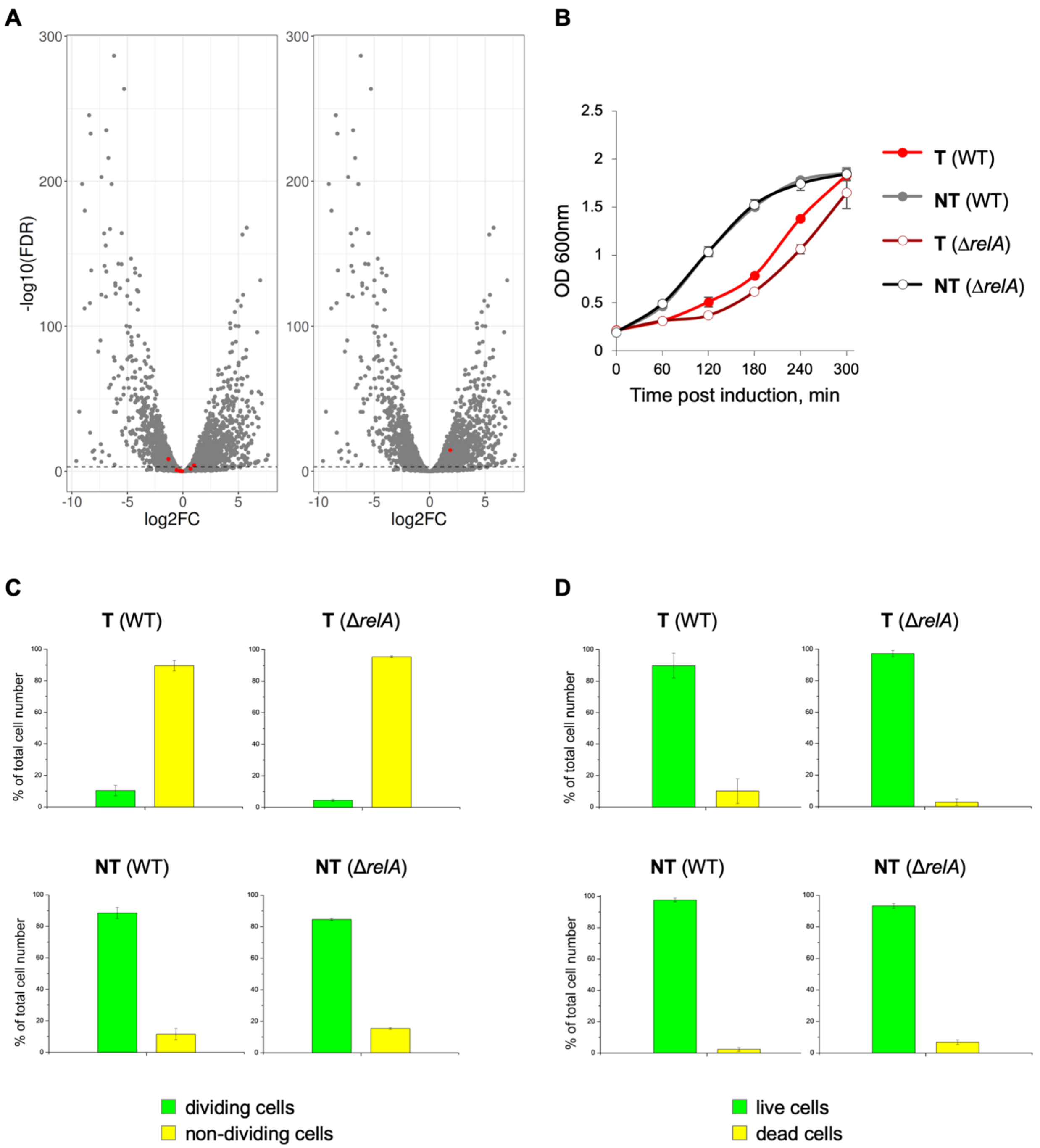

Fig. S17. Cas13a activation in targeting cells does not induce RelA-dependent stringent response. (**A**) Analysis of differential gene expression in *E. coli* cells upon Cas13a RNA-targeting did not reveal gene expression pattern characteristic of stringent response. RNA targeting does not reshape expression of genes up- (left panel) or downregulated (right panel) during stringent response conditions (*17*) (stringent-response dependent genes are taken from Ref. 17 and highlighted by red color). (**B**) Targeting of RFP mRNA by LshCas13a leads to growth suppression in targeting (T, dark red) cells lacking RelA (Δ*relA* strain) similar to targeting cells expressing RelA (WT, shown in red); no such effect is observed in nontargeting *ΔrelA* (dark grey) and WT

(light grey) controls. OD_{600} values represent a mean ± SEM of three biological replicates at each time point. (**C**) Time-lapse microscopy revealed no effect of *relA* deletion on the portion of dividing cells in targeting cultures. Similar to wild type cells, majority of cells lacking RelA stop to divide upon RNA targeting. Bar graph shows mean percentages (± SEM) of dividing and non-dividing cells from the total number of cells counted for targeting (top graph) and nontargeting (bottom graph) samples, obtained from three biological replicates. (**D**) The membrane-impermeable dye YOYO-1 staining for the detection of dead cells showed that *relA* deletion does not increase the number of dead cells upon RNA targeting suggesting RelA-independent mechanism of dormancy.

Table S1.
tRNA cleavage sites revealed by RNA-seq in wild-type *E. coli* cells expressing LshCas13a upon RFP mRNA targeting (related to Fig. 2H).

Seq ID	Position	Strand	logFC	PValue.adj	Matched feature, gene
NC_000913.3	781181	+	7.15895559514473	6.91866110521177E-55	*lysY*
NC_000913.3	780876	+	6.36128002000647	1.84947255616146E-50	*lysW*
NC_000913.3	2729410	-	7.35611610949685	6.96305307286096E-50	*gltW*
NC_000913.3	4175421	+	6.77232288795697	3.56992654922837E-49	*thrU*
NC_000913.3	780587	+	6.47494075209259	1.07898866135737E-47	*lysT*
NC_000913.3	781402	+	6.4174456716735	1.03867438464741E-45	*lysZ*
NC_000913.3	780588	+	8.10268574320133	3.14980499727998E-45	*lysT*
NC_000913.3	780877	+	8.10306795517297	4.48090625852247E-42	*lysW*
NC_000913.3	4209809	+	5.73675506962861	5.12589005119606E-41	*gltV*
NC_000913.3	3943470	+	6.15655839481644	6.04917254713136E-40	*gltU*
NC_000913.3	4175422	+	4.70063823385595	6.04917254713136E-40	*thrU*
NC_000913.3	4168407	+	5.1048247594494	7.5888583029461E-38	*gltT*
NC_000913.3	781403	+	8.08153117275539	7.25159717928984E-37	*lysZ*
NC_000913.3	696799	-	7.25923518046918	1.62548345087711E-35	*glnW*
NC_000913.3	696908	-	7.30706075691888	3.00714263689625E-32	*glnU*
NC_000913.3	697014	-	4.25183199397708	4.50341765107348E-30	*leuW*
NC_000913.3	3708658	-	4.6209712254373	2.35249043556951E-19	*proK*
NC_000913.3	696472	-	5.8747196773628	8.83022273617196E-15	*glnX*
NC_000913.3	696584	-	5.59142803493453	4.18000833955816E-13	*glnV*

Table S2.
tRNA cleavage sites revealed by RNA-seq in Δ10 *E. coli* cells expressing LshCas13a upon RFP mRNA targeting (related to Fig. 3D).

Seq ID	Position	Strand	logFC	PValue.adj	Matched feature, gene
NC_000913.3	262905	+	5.04757696827469	1.52032887669118E-28	*thrW*
NC_000913.3	564757	+	4.63110421504942	1.26108845053698E-29	*argU*
NC_000913.3	780587	+	4.5663397955008	1.51498453810283E-33	*lysT*
NC_000913.3	780588	+	5.38248322270425	1.03100693998586E-54	*lysT*
NC_000913.3	780877	+	4.84062921287924	6.10954114070889E-39	*lysW*
NC_000913.3	781181	+	5.24807673407979	1.22527050712166E-53	*lysY*
NC_000913.3	781403	+	5.18485630519495	3.58474846835013E-50	*lysZ*
NC_000913.3	781611	+	5.73658706150962	2.15810605858204E-37	*lysQ*
NC_000913.3	2520964	+	4.50037020004344	1.52271794330762E-31	*valU*
NC_000913.3	2521287	+	5.00373431779725	3.10021908445095E-31	*lysV*
NC_000913.3	3836256	+	4.37978187613894	4.46707697166585E-20	*selC*
NC_000913.3	3943470	+	5.24855541884814	3.58474846835013E-50	*gltU*
NC_000913.3	4168407	+	4.91333931544101	2.13289862042611E-45	*gltT*
NC_000913.3	4175421	+	6.77366197513059	2.19765362102023E-71	*thrU*
NC_000913.3	4175422	+	5.98040559458023	5.33206859695168E-57	*thrU*
NC_000913.3	4175706	+	4.24001203483477	4.70342463163678E-28	*glyT*
NC_000913.3	4209809	+	5.3150584663947	2.14290647075459E-54	*gltV*
NC_000913.3	696438	-	5.98315405144837	1.42355936918166E-24	*glnX*
NC_000913.3	696471	-	7.083688015588	1.55036471920295E-59	*glnX*
NC_000913.3	696550	-	5.90215218777287	3.82053403754773E-27	*glnV*
NC_000913.3	696583	-	6.91431203669071	7.69713502742078E-69	*glnV*
NC_000913.3	696797	-	5.1750274819562	2.57285120937203E-50	*glnW*
NC_000913.3	696875	-	4.53118948261685	9.22196054547485E-22	*glnU*
NC_000913.3	696906	-	4.9922981894158	6.90921462473473E-47	*glnU*
NC_000913.3	696907	-	4.18448856321443	4.6700864808674E-27	*glnU*
NC_000913.3	2729409	-	4.58833062852572	2.08894029231763E-41	*gltW*

Table S3.
Toxin-antitoxin systems encoded in *Leptotrichia shahii*.

Origin	Coordinates	Strand	Protein ID	Description
Genome	326082…326368	-	WP_026231269.1	type II toxin-antitoxin system YafQ family toxin
Genome	326368…326646	-	WP_018450943.1	type II toxin-antitoxin system RelB/DinJ family antitoxin
Genome	425849…426100	+	WP_018450842.1	type II toxin-antitoxin system AbrB/MazE/SpoVT family antitoxin
Genome	426094…426423	+	WP_018450841.1	type II toxin-antitoxin system PemK/MazF family toxin
Genome	1080797…1081051	+	WP_018450010.1	type II toxin-antitoxin system Phd/YefM family antitoxin
Genome	1081044…1081298	+	WP_018450009.1	type II toxin-antitoxin system Txe/YoeB family toxin
Genome	1181321…1181578	-	WP_018449908.1	type II toxin-antitoxin system Txe/YoeB family toxin
Genome	1181578…1181835	-	WP_018449907.1	type II toxin-antitoxin system Phd/YefM family antitoxin
Plasmid	1544…1759	+	WP_006806162.1	antitoxin
Plasmid	1744…2016	+	WP_018450732.1	type II toxin-antitoxin system RelE/ParE family toxin
Plasmid	13054…13269	+	WP_149201970.1	antitoxin
Plasmid	13254…13526	+	WP_149201971.1	type II toxin-antitoxin system RelE/ParE family toxin

Putative toxin-antitoxin systems encoded in *Leptotrichia shahii* genome (RefSeq NZ_AP019827.1) and the resident plasmid (RefSeq NZ_AP019828.1) presented in TADB 3.0: an updated database of bacterial toxin–antitoxin loci and associated mobile genetic elements (*13*).

Table S4.
tRNA cleavage sites revealed by RNA-seq in M13-infected *E. coli* cells expressing LshCas13a upon targeting phage transcript (related to Fig. 4H).

Seq ID	Position	Strand	logFC	PValue.adj	Matched feature, gene
NC_000913.3	781181	+	10.9406594470345	2.23493623221352E-78	*lysY*
NC_000913.3	780877	+	9.34390377567968	1.43300576561857E-72	*lysW*
NC_000913.3	781402	+	8.12486245646455	2.07520329408417E-71	*lysZ*
NC_000913.3	780588	+	9.63832365082055	7.4113610071658E-66	*lysT*
NC_000913.3	780876	+	7.89826595951874	1.04829425944103E-62	*lysW*
NC_000913.3	781403	+	10.0158358742056	1.57905051939578E-60	*lysZ*
NC_000913.3	2729409	-	7.17763795666644	8.76845727875601E-60	*gltW*
NC_000913.3	696797	-	6.48200334358892	3.46766307887245E-59	*glnW*
NC_000913.3	780587	+	7.6041655690818	6.059195027361E-57	*lysT*
NC_000913.3	3943470	+	7.55976774618007	8.92057779408653E-57	*gltU*
NC_000913.3	696906	-	6.43475232718212	8.60881675424876E-56	*glnU*
NC_000913.3	4175421	+	6.01178488307341	1.40740891040437E-51	*thrU*
NC_000913.3	4209809	+	6.45679135962629	3.0834298540971E-50	*gltV*
NC_000913.3	4168407	+	6.45777953635544	2.4684221162918E-46	*gltT*
NC_000913.3	4175422	+	4.91005006113337	6.31983193982276E-42	*thrU*
NC_000913.3	781409	+	5.0111783214955	1.58826746863603E-31	*lysZ*
NC_000913.3	4037335	+	4.11988967559717	2.23133327574455E-21	*alaT*

Table S5. Plasmids used in this study.

Name	Description	Source
**pC008**	pBR322 with tetR-inducible RFP and a *bla* gene	(*2*)
**pC002**	LshCas13a locus into PACYC184, the native CRISPR array contains a spacer1 (AGCGGTCTGGGTACCTTCGTACGGACGACCT) matching an engineered protospacer in M13. Referred to as M13 targeting variant in Fig. 4	(*2*)
**pC003**	LshCas13a locus into PACYC184 with BsaI sites for spacer cloning[a]	(*2*)
**pC003_RFP1**	pACYC184 with LshCas13a locus containing a spacer matching RFP mRNA (CGGTCTGGGTACCTTCGTACGGACGACCTTC). Referred to as crRNA1 in Fig. 3B, C and fig. S8	(*2*)
**pC003_RFP2**	pACYC184 with LshCas13a locus containing a spacer matching RFP mRNA (TACGGACGACCTTCACCTTCACCTTCGATTT). Referred to as crRNA2 in Fig. 3B, C and fig. S8	This study
**pC003_RFP3**	pACYC184 with LshCas13a locus containing a spacer matching RFP mRNA (AGCGGTCTGGGTACCTTCGTACGGACGACCT). Referred to as crRNA2 in Fig. 3B, C and fig. S8	This study
**pC003_Sp1**	pACYC184 with LshCas13a locus containing a spacer (AGCGGTCTGGGTACCTTCGTACGGACGACCT) that does not match *E. coli* genome or the plasmid sequences. Referred to as nontargeting control in RFP mRNA target experiments.	This study
**pC003_cpMS2**	pACYC184 with LshCas13a locus containing a spacer (GAGACCTTGCATTGCCTTAACAATAAGCTCG) targeting the MS2 *cp* gene, not matching any *E. coli*, M13, or the plasmid sequences. Referred to as nontargeting control in the M13 phage experiments.	(*2*)
**pC003^{R597A}_RFP**	pC003_RFP1 with R597A mutation in LshCas13a	This study
**pC003^{H602A}_RFP**	pC003_RFP1 with H602A mutation in LshCas13a	This study
**pC003^{R1278A}_RFP**	pC003_RFP1 with R1278A mutation in LshCas13a	This study
**pC003^{H1283A}_RFP**	pC003_RFP1 with H1283A mutation in LshCas13a	This study
**pET28_LshCas13a**	For isolation of LshCas13a protein	This study
**pET28_LshCas13a^{H602A}**	For isolation of LshCas13a^{H602A} mutant protein	This study
**pC008_tRNA^{cleavable}**	For overexpression of tRNA^{lys}, tRNA^{gln}, tRNA^{glu}, and tRNA^{leu} (most strongly affected by target-activated Cas13a)[b, c]	This study
**pC008_tRNA^{uncleavable}**	For overexpression of tRNA^{ser}, tRNA^{pro}, tRNA^{asp}, and tRNA^{trp} (unaffected by target-activated Cas13a)[b, d]	This study

[a]There is a premature stop in Cas2 protein encoded in this plasmid. As Cas1 and Cas2 are not required for RNA targeting, only *cas13a* gene is shown in the plasmid schematic for pC003 derivatives in Fig. 1B for simplicity.

Synthetic gBlocks sequences used for construction of pC008_tRNA^{cleavable} and pC008_tRNA^{uncleavable} plasmids

[b]gBlock for cloning lipoprotein promoter cassette:
AACGCTG**CCCGAG**AGATAAAAAAAATCCTTAGCTTTCGCTAAGGAT**gctgagg***TGGTGGGTCGTGCAGGATTCGAACCTGCGACCAATTGATTAAAAGTCAACTGCTCTACCAACTGAGCTAACGACCC***actagt**AGCGTTACAAGTATTACACAAAGTTTTTATGTTGAGAATATTTTTTTGATGGGGCGCCACTTATTTTTGATCGTTCGCTCAAAGAAGCGGCG**CCCGAG**ATGCGCC
Complement sequences of lipoprotein promoter, tRNA^{lys} gene, and *rrnC* terminator are single-underlined, shown in italic type, and double-underlined, respectively. Cloning AvaI sites are shown in bold type. Internal BbvCI and SpeI sites shown in bold type and lowercase can be used for subsequent cloning.

[c]gBlock for cloning tRNAs affected by target-activated Cas13a:
GAGTGCAGC**gctgagg**TGGTGGGTCGTGCAGGATTCGAACCTGCGACCAATTGATTAAAAGTCAACTGCTCTACCAACTGAGCTAACGACCCACTTTTTCGTTGCTTTCTTGAATAAATTGGCTGGGGTACGAGGATTCGAACCTCGGAATGCCGGAATCAGAATCCGGTGCCTTACCGCTTGGCGATACCCCAACAAACCAGCAAGTGGCGTCCCCTAGGGGATTCGAACCCCTGTTACCGCCGTGAAAGGGCGGTGTCCTGGGCCTCTAGACGAAGGGGACGTATCAGTCTGCTTCGCAAGACGCCTTGCTTTCTGGTGAATGGTGCGGGAGGCGAGACTTGAACTCGCACACCTTGCGGCGCCAGAACCTAAATCTGGTGCGTCTACCAATTTCGCCACTCCCGC**actagt**AGCTATGAC
Complement sequences of genes coding for tRNA^{lys}, tRNA^{gln}, tRNA^{glu}, and tRNA^{leu} are underlined. Cloning BbvCI and SpeI sites are shown in bold type and lowercase.

[d]gBlock for cloning tRNAs unaffected by target-activated Cas13a:
GAGACGTA**gctgagg**TGGCAGGGGCGGAGAGACTCGAACTCCCAACACCCGGTTTTGGAGACCGGTGCTCTACCAATTGAACTACGCCCCTAATTAGGGTGGCGGAACGGACGGGACTCGAACCCGCGACCCCCTGCGTGACAGGCAGGTATTCTAACCGACTGAACTACCGCTCCACCGAATTCTTTCTTGGGATTTTTGGTCGGCACGAGAGGATTTGAACCTCCGACCCCCGACACCCCATGACGGTGCGCTACCAGGCTGCGCTACGTGCCGACTCGTGGCTGCTACTTCTTTAAATATGGCGGTGAGGGGGGGATTCGAACCCCCGATACGTTGCCGTATACACACTTTCCAGGCGTGCTCCTTCAGCCACTCGGACACCTCACC**actagt**AGCTATGAC
Complement sequences of genes coding for tRNA^{ser}, tRNA^{pro}, tRNA^{asp}, and tRNA^{trp} are underlined. Cloning BbvCI and SpeI sites are shown in bold type and lowercase.

Table S6.
Oligonucleotides used in this study.

Name	Description	Sequence (5' to 3')
**bla_PE**	Primer extension, *bla* mRNA	TGGGTGAGCAAAAACAGGAAGG
**rpmH_PE**	Primer extension, *rpmH* mRNA	TAGCCATACGAGCACGGAAGC
**lys_PE**	Primer extension, tRNA^{lys}	GGTGGGTCGTGCAGGATTC
**gln_PE**	Primer extension, tRNA^{gln}	TGGCTGGGGTACGAGGATT
**leu_RE**	Primer extension, tRNA^{leu}	GGTGCGGGAGGCGAGACTTGA
**ala_PE**	Primer extension, tRNA^{ala}	GGGATCGAACCGCAGACC
**R597A_F**	HEPN mutagenesis	CAAAGATAGGAACAAATGAAGCAAACAGGATATTACATGCG
**R597A_R**	HEPN mutagenesis	CGCATGTAATATCCTGTTTGCTTCATTTGTTCCTATCTTTG
**H602A_F**	HEPN mutagenesis	ATGAAAGAAACAGGATATTAGCTGCGATTAGCAAGGAAAGAG
**H602A_R**	HEPN mutagenesis	CTCTTTCCTTGCTAATCGCAGCTAATATCCTGTTTCTTTCAT
**R1278A_F**	HEPN mutagenesis	GAAAATGAAAGTATTGCAAACTATATTTCACA
**R1278A_R**	HEPN mutagenesis	TGTGAAATATAGTTTGCAATACTTTCATTTTC
**H1283A_F**	HEPN mutagenesis	GTATTAGAAACTATATTTCAGCTTTCTATATTGTAAGAAATCC
**H1283A_R**	HEPN mutagenesis	GGATTTCTTACAATATAGAAAGCTGAAATATAGTTTCTAATAC
**RFP_crRNA_probe**	Northern blot hybridization	GAAGGTACCCAGACCGGTTTTAGTCCC
**lysT-rev-fam_probe**	Northern blot hybridization	GTCGTGCAGGATTCGAACCT-3' 6-FAM (Fluorescein)
**glnV-rev-fam-probe**	Northern blot hybridization	AGGATTCGAACCTCGGAATGC-3' 6-FAM (Fluorescein)
**leuW-rev-fam-probe**	Northern blot hybridization	TGCGGGAGGCGAGACTT-3' 6-FAM (Fluorescein)
**crRNA_template**	DNA template for *in vitro* transcription crRNA*	GGTTTTCCCAGTCACGACGTTGTAAACGACGGCCAGTGAATTC<br>taatacgactcactata**GGCCACCCCAATATCGAAGGGGACTAAAACTA**<br>**GATTGCTGTTCTACCAAGTAATCCAT**ATT
**targetRNA_template**	DNA template for *in vitro* transcription target RNA*	taatacgactcactata**GGGACATTAAGGCGAAAGTAAAAGATTATCCGAGTT**<br>**ATGCAGCCGGAAATATACAATATGGATTACTTGGTAGAACAGCAAT**<br>**CTACTTAAATACGTTTCGCGTTATGAATACAAAAACGGCA**TTGAAGA<br>TTTGAAGAAAGCGCAGTTTTATTTGAATGATTTTT
**tRNA^{lys}**	*in vitro* cleavage	GGGUCGUUAGCUCAGUUGGUAGAGCAGUUGACUUUUAAUCAA<br>UUGGUCGCAGGUUCGAAUCCUGCACGACCCACCA

*T7 promoter is shown in lowercase letters, PCR primers are underlined, RNA-coding region is highlighted in bold type.
